# Hybrid density functional study of strain-engineered III–V semiconductors and monolayer InSe

**DOI:** 10.1039/d6ra06960f

**Published:** 2026-09-14

**Authors:** Md. Tanvir Ahmed, Maahi Sabah, Md. Saiful Islam, Mahabuba Rahman Shanta, Md. Hafijur Rahman, Md. Ashraf Ali, Ruma Parvin, Saleh Hasan Naqib, Md. Shahajan Ali

**Affiliations:** a Department of Physics, Pabna University of Science and Technology Pabna-6600 Bangladesh msali@pust.ac.bd; b Theoretical and Experimental Advanced Materials Science Lab (TEAMS), Pabna University of Science and Technology Pabna-6600 Bangladesh; c Department of Physics, University of Rajshahi Rajshahi-6205 Bangladesh; d Department of Physics, Chittagong University of Engineering and Technology Chattogram-4349 Bangladesh

## Abstract

Accurate prediction of strain-dependent semiconductor properties requires reliable exchange–correlation treatments and a unified understanding of bulk and two-dimensional materials. Here, we investigate strain-engineered bulk AlP, AlSb, and monolayer InSe under biaxial strains from −6% to +6% using the Perdew–Burke–Ernzerhof (PBE) and Heyd–Scuseria–Ernzerhof (HSE06) functionals. HSE06 significantly improves structural accuracy, reducing the mean absolute deviation in lattice parameters from 1.47% to 0.39%. Biaxial strain lowers the symmetry of bulk AlP and AlSb from cubic zinc blende to tetragonal structures, whereas monolayer InSe retains its hexagonal symmetry. Phonon spectra and *ab initio* molecular dynamics confirm the stability of all materials, although monolayer InSe develops small imaginary phonon frequencies near the *Γ*-point under compressive strain. Thermodynamic properties remain insensitive to strain. Compressive strain increases the elastic stiffness of all three materials, whereas tensile strain reduces it. Elastic anisotropy increases under compression and decreases under tension in bulk AlP and AlSb, while monolayer InSe remains isotropic throughout the investigated strain range. HSE06 reproduces the experimental band gaps, revealing a tensile-induced indirect-to-direct band-gap transition in AlSb and a band-gap tunability of approximately 1.69 eV in monolayer InSe. Tensile strain redshifts the absorption edge, highlighting the potential of these materials for strain-engineered optoelectronic and flexible electronic devices.

## Abstract 2

Biaxial strain effectively tunes the structural, electronic, mechanical, and optical properties of bulk AlP, bulk AlSb and monolayer InSe, enabling an indirect-to-direct transition in AlSb and strong band-gap modulation in InSe.

## Graphical abstract

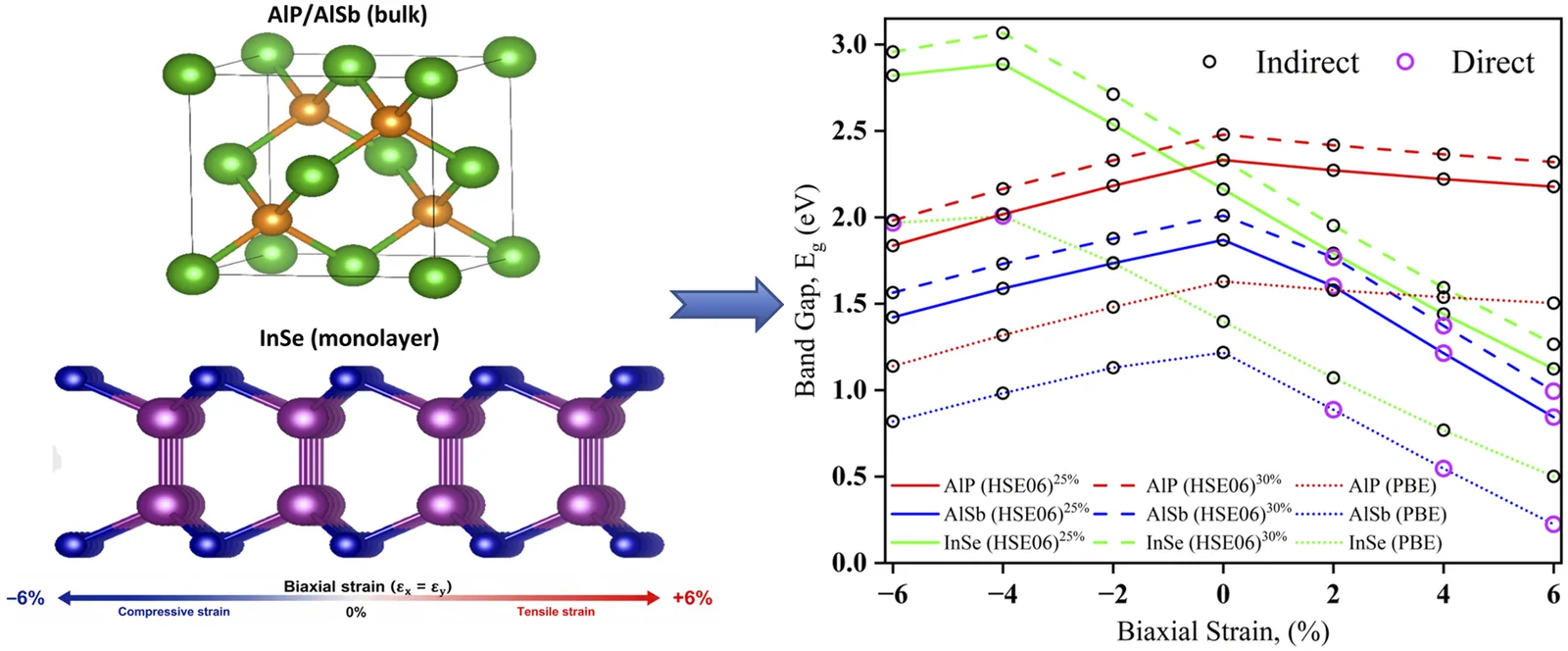

## 1 Introduction

The increasing demand for miniaturised, high-performance electronic and optoelectronic devices has intensified the search for semiconductors with highly tuneable physical properties. III–V semiconductors, such as aluminium phosphide (AlP) and aluminium antimonide (AlSb), are widely recognized for their wide band gaps, high carrier mobility, and excellent thermal stability, making them promising candidates for high-frequency and high-power electronic applications.^1–4^ In parallel, two-dimensional (2D) semiconductors such as indium selenide (InSe) have attracted significant attention because of their atomic-scale thickness, strong quantum confinement, and highly strain-sensitive electronic structures.^5–7^ Owing to their distinct dimensionalities and bonding characteristics, bulk III-V and low-dimensional semiconductors can exhibit fundamentally different responses to external lattice deformation, offering opportunities for strain-tuneable electronic and optoelectronic functionalities.

Strain engineering has emerged as an effective strategy for modulating semiconductor properties through controlled lattice deformation. By modifying interatomic distances and orbital overlap, biaxial strain can significantly alter band dispersion, carrier transport behaviour, optical transitions, and mechanical response.^8–11^ Previous theoretical and experimental studies have demonstrated substantial strain-induced tunability in InSe, including strain-dependent band-gap evolution, changes in band-gap character, and strong piezoresistive behaviour. However, previous theoretical studies have primarily relied on semilocal PBE calculations, while the experimental work focused on ultrathin InSe devices.^12–14^ A hybrid-functional assessment of the strain-dependent properties of monolayer InSe, particularly the influence of exchange–correlation treatment on its electronic structure, remains limited. Systematic investigations of strain-engineered bulk AlP and bulk AlSb also remain comparatively limited, particularly within hybrid-functional frameworks capable of accurately describing strain-dependent electronic properties. In contrast, strain-dependent electronic properties have been investigated in low-dimensional AlP and AlSb, including strain-induced direct-indirect band-gap transitions in tetragonal AlP and electronic anisotropy and band inversion in double-layer honeycomb AlSb.^15,16^ Furthermore, most previous studies focus on isolated material systems and often rely on conventional generalized-gradient approximations, making direct comparisons between bulk III–V and low-dimensional semiconductors under equivalent strain conditions still scarce.^17–20^ Consequently, a unified understanding of how dimensionality and exchange–correlation treatment jointly influence strain-driven structural stability, mechanical anisotropy, thermodynamic behaviour, optical response, and electronic behaviour remains lacking.

Another important challenge lies in the limitations of conventional density functional theory (DFT) methods for accurately describing semiconductor electronic structures. The Perdew–Burke–Ernzerhof (PBE) functional, although computationally efficient, is well known for underestimating band gaps and occasionally misclassifying band characteristics.^21,22^ For example, AlP, experimentally known as an indirect-gap semiconductor with a band gap near 2.50 eV, is frequently predicted by PBE to exhibit a significantly smaller gap and altered electronic features.^23–25^ Hybrid functionals such as Heyd–Scuseria–Ernzerhof (HSE06) substantially improve predictive accuracy by incorporating a fraction of exact exchange. Previous studies on III–V semiconductors have shown that HSE06 generally provides much better agreement with experimental electronic properties than standard generalized-gradient approximations.^26–29^

Motivated by these unresolved issues, this work presents a systematic first-principles investigation of strain-engineered bulk AlP, bulk AlSb, and monolayer InSe using both PBE and HSE06 functionals. AlP and AlSb are selected as representative bulk III–V semiconductors with distinct bonding strength and strain sensitivity, while monolayer InSe serves as a representative low-dimensional semiconductor with highly tuneable electronic behaviour. By combining these systems within a unified computational framework, this study enables direct comparison of dimensionality-dependent strain responses under equivalent biaxial strain conditions (±2%, ±4%, and ±6%).^30^ In addition to structural stability and mechanical behaviour, we examine strain-dependent electronic, optical, and thermodynamic properties, including orbital-resolved electronic features. Particular emphasis is placed on understanding how strain and exchange–correlation treatment govern band-gap evolution, anisotropic mechanical response, thermodynamic behaviour, and optical absorption. To the best of our knowledge, a unified hybrid-functional investigation combining structural stability, mechanical anisotropy, thermodynamic properties, optical response, and electronic behaviour across strain-engineered bulk AlP, bulk AlSb, and monolayer InSe has not yet been reported.

Understanding these coupled strain-dependent responses is important for accurately predicting and tuning the electronic, optical, and transport behaviour of semiconductors under lattice deformation. By systematically comparing bulk III–V and low-dimensional semiconductor systems within a unified PBE-HSE06 framework, this work provides a consistent understanding of how dimensionality and exchange–correlation treatment influence strain-dependent material properties relevant to tuneable optoelectronic applications.

## 2 Computational methods

First-principles calculations were performed using the Vienna *Ab initio* Simulation Package (VASP) employing the projector augmented-wave (PAW) method.^31,32^ Exchange–correlation effects were described using both the Perdew–Burke–Ernzerhof (PBE) generalized-gradient approximation and the screened hybrid Heyd–Scuseria–Ernzerhof (HSE06) functional.^33,34^ Structural, mechanical, electronic, and optical properties were evaluated using both functionals, while phonon, thermodynamic properties, and *ab initio* molecular dynamics (AIMD) simulations were performed using the PBE functional due to the substantially higher computational cost associated with hybrid-functional lattice-dynamical calculations. Although HSE06 may quantitatively modify phonon frequencies and the finite-temperature potential-energy surface, PBE was used to assess qualitative dynamical and thermal stability. Phonon stability was evaluated from the absence of imaginary modes rather than precise phonon frequencies, while AIMD was used to assess structural integrity rather than precise energetic properties. The valence electron configurations adopted for the PAW pseudopotentials were 3s^2^3p^1^ for Al, 3s^2^3p^3^ for P, 5s^2^5p^3^ for Sb, 5s^2^5p^1^ for In, and 4s^2^4p^4^ for Se. A plane-wave cutoff energy of 500 eV was employed throughout all calculations. Brillouin-zone integrations utilized *Γ*-centred *k*-point meshes of 11 × 11 × 11 for bulk AlP and AlSb, and 13 × 13 × 1 for monolayer InSe during structural optimization and elastic constant calculations. To eliminate spurious interactions between periodically repeated layers in the monolayer InSe system, a vacuum spacing of 25 Å was introduced along the out-of-plane direction. The electronic self-consistency and ionic relaxation convergence criteria were set to 1 × 10^−6^ eV and 0.01 eV Å^−1^, respectively, with a Gaussian smearing width of 0.05 eV applied throughout.

Biaxial strain was systematically introduced by uniformly scaling the in-plane lattice vectors, including both tensile and compressive strain states of ±2%, ±4%, and ±6%.^35^ Ionic positions were subsequently relaxed under fixed strained lattice conditions. Elastic constants were calculated using the energy-strain method, in which small lattice deformations were applied to the relaxed structures and the resulting total-energy variations were fitted using second-order polynomial functions.^36^ For monolayer InSe, the same energy-strain formalism was applied to the in-plane deformation modes, with the elastic response normalized by the equilibrium in-plane area, yielding two-dimensional elastic constants in Nm^−1^, consistent with established treatments of 2D materials.^37^ The elastic moduli and Poisson's ratio were calculated from the corresponding elastic constants *C*_*ij*_.^38,39^ VASPKIT was employed to generate the strain configurations and extract the elastic tensors.^40^ Directional elastic properties were evaluated from the elastic compliance tensor using spherical-coordinate transformations.

Phonon dispersion relations, phonon density of states (DOS), and thermodynamics properties were calculated using the finite-displacement method implemented in the Phonopy package.^41^ For phonon calculations, 2 × 2 × 2 supercells were used for bulk AlP and AlSb, while a 4 × 4 × 1 supercell was employed for monolayer InSe, with each supercell containing 64 atoms. Corresponding *Γ*-centred *k*-point meshes of 7 × 7 × 7 for the bulk systems and 5 × 5 × 1 for monolayer InSe were adopted to ensure convergence of phonon frequencies and minimize artificial periodic–image interactions. Finite-temperature stability was further examined using AIMD simulations within the canonical (NVT) ensemble employing a Nosé–Hoover thermostat at 300 K for 5 ps (1 fs timestep), with *Γ*-point sampling on the same supercells as phonon calculations.^42^

Hybrid-functional calculations were performed using the HSE06 functional with a screening parameter of 0.2 Å^−1^. An exact-exchange mixing parameter of 25% was used for structural and mechanical, and optical properties calculations, while both 25% and 30% exact-exchange fractions were examined for the electronic properties to evaluate the sensitivity of strain-dependent band structures to hybrid-functional mixing.^43^ High-symmetry paths for both electronic and phonon band structures were generated using the SeeK-path package.^44^ Frequency-dependent dielectric functions and optical absorption spectra were calculated within the independent-particle approximation using the linear optical routines implemented in VASP.

## 3 Results and discussion

### 3.1 Structural properties and stability

**Fig. 1:**
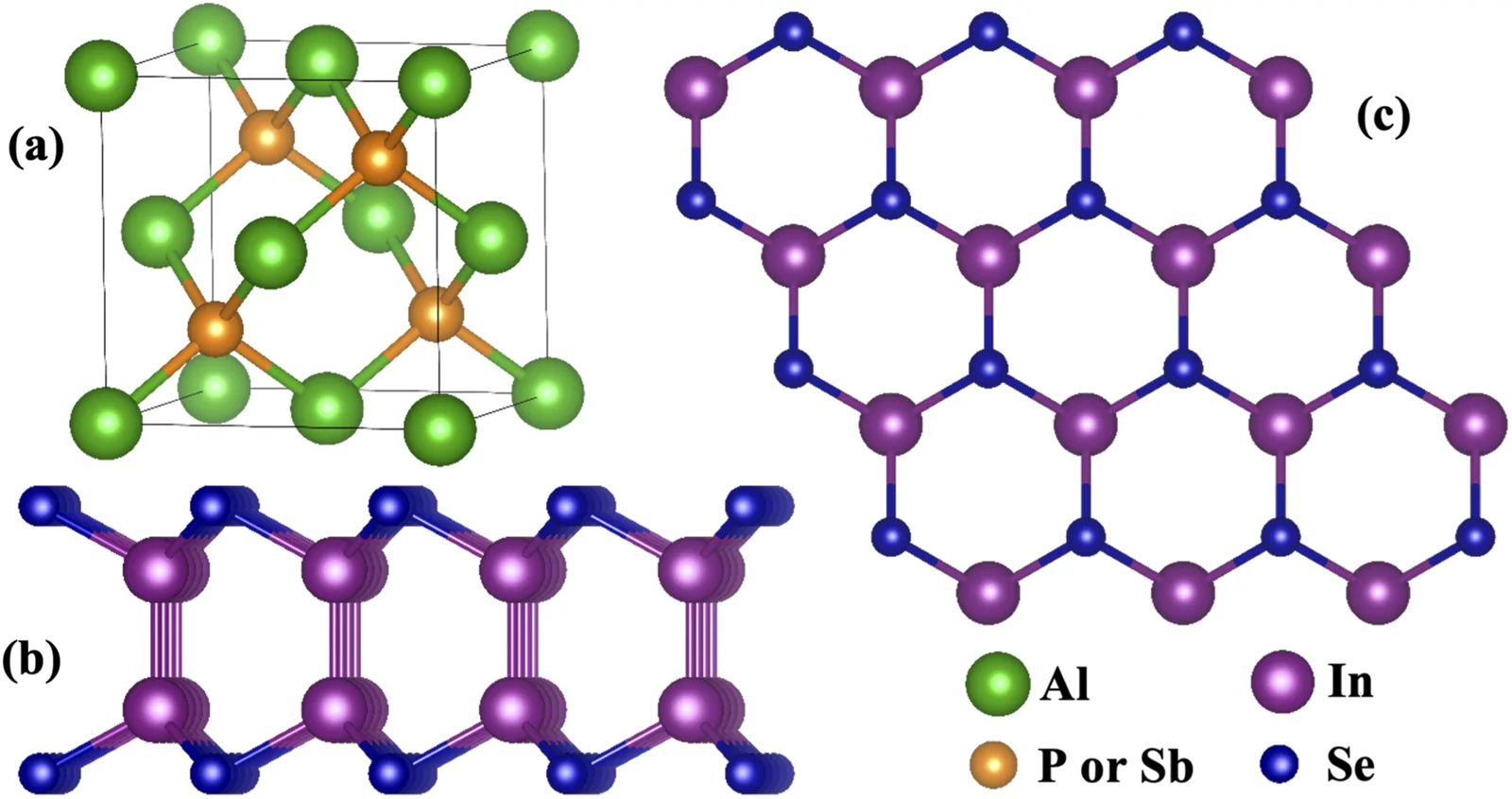
| Relaxed unstrained crystal structures of (a) bulk AlP and AlSb, and monolayer InSe shown in (b) side view and (c) top view.

The optimized crystal structures are shown in Fig. 1. The equilibrium structures of AlP, AlSb, and monolayer InSe were optimized using both the PBE and hybrid HSE06 functionals, with the corresponding structural parameters summarized in Table 1. The strain-dependent structural data are provided in SI Table S1. PBE systematically overestimates the lattice parameters relative to experimental values, yielding a mean absolute deviation of approximately 1.47%. Inclusion of exact exchange in HSE06 significantly improves the structural accuracy, reducing the mean absolute deviation to approximately 0.39%, corresponding to a reduction of about 73%. The improved agreement obtained with HSE06 reflects the reduced self-interaction error and the more accurate description of bonding interactions provided by hybrid exchange. Similar improvements in structural and electronic accuracy using hybrid functionals have previously been reported for III–V semiconductors.^45^

**Table 1:** Optimized structural parameters, bond lengths (*d*), volume per formula unit (*V*), and formation energies per formula unit (*E*_f_) of unstrained bulk AlP, AlSb, and monolayer InSe calculated using PBE and HSE06 functionals. Experimental values are included for comparison where available

Material	Functional	*a* (Å)	*a* (Expt.) (Å)	Δ*a* %	*c* (Å)	*d* (Å)	*V* (Å^3^ per f.u.)	*E*_f_ (eV per f.u.)	*E*_f_ (Expt.) (eV per f.u.)
AlP	PBE	5.506	5.467 (ref. 46)	0.71	—	2.384_Al–P_	41.74	−1.261	−1.22 (ref. 49)
	HSE06	5.473		0.11	—	2.370_Al–P_	40.98	−1.458	
AlSb	PBE	6.235	6.136 (ref. 47)	1.61	—	2.699_Al–Sb_	60.59	−0.336	−0.59 (ref. 50)
	HSE06	6.149		0.21	—	2.663_Al–Sb_	58.13	−0.614	
InSe	PBE	4.083	4.000 (ref. 48)	2.08	25.330	2.682_In–Se_	182.81	−1.049	—
						2.825_In–In_			
	HSE06	4.034		0.85	25.033	2.645_In–Se_	176.42	−1.271	
						2.794_In–In_			

HSE06 additionally predicts smaller equilibrium cell volumes and stronger binding energies than PBE. Relative to PBE, the equilibrium volume per formula unit decreases by approximately 1.8% for AlP, 4.1% for AlSb, and 3.5% for monolayer InSe. Similarly, the calculated formation energies become more exothermic by an average of approximately 0.23 eV per f.u., indicating enhanced stabilization of the equilibrium structures within the hybrid-functional framework. HSE06 also produces a noticeable contraction of the internal bond lengths in monolayer InSe, whereas AlP and AlSb exhibit comparatively smaller and material-dependent bond shortening, as summarized in Table 1.

Biaxial strain reduces the cubic *F*4̄3*m* (216, Td) symmetry of AlP and AlSb to tetragonal *I*4̄*m*2 (119, C4v), removing the three-fold rotational symmetry together with several mirror and rotoinversion symmetries, while preserving the four-fold rotational axis and vertical mirror symmetries. In contrast, monolayer InSe retains its hexagonal *P*6̄*m*2 (187) symmetry under the same strain conditions. This strain-induced symmetry reduction introduces pronounced anisotropy in the III–V compounds, which subsequently influences their electronic band structure and mechanical response. The complete strain-dependent structural parameters are listed in SI Table S1.

**Fig. 2:**
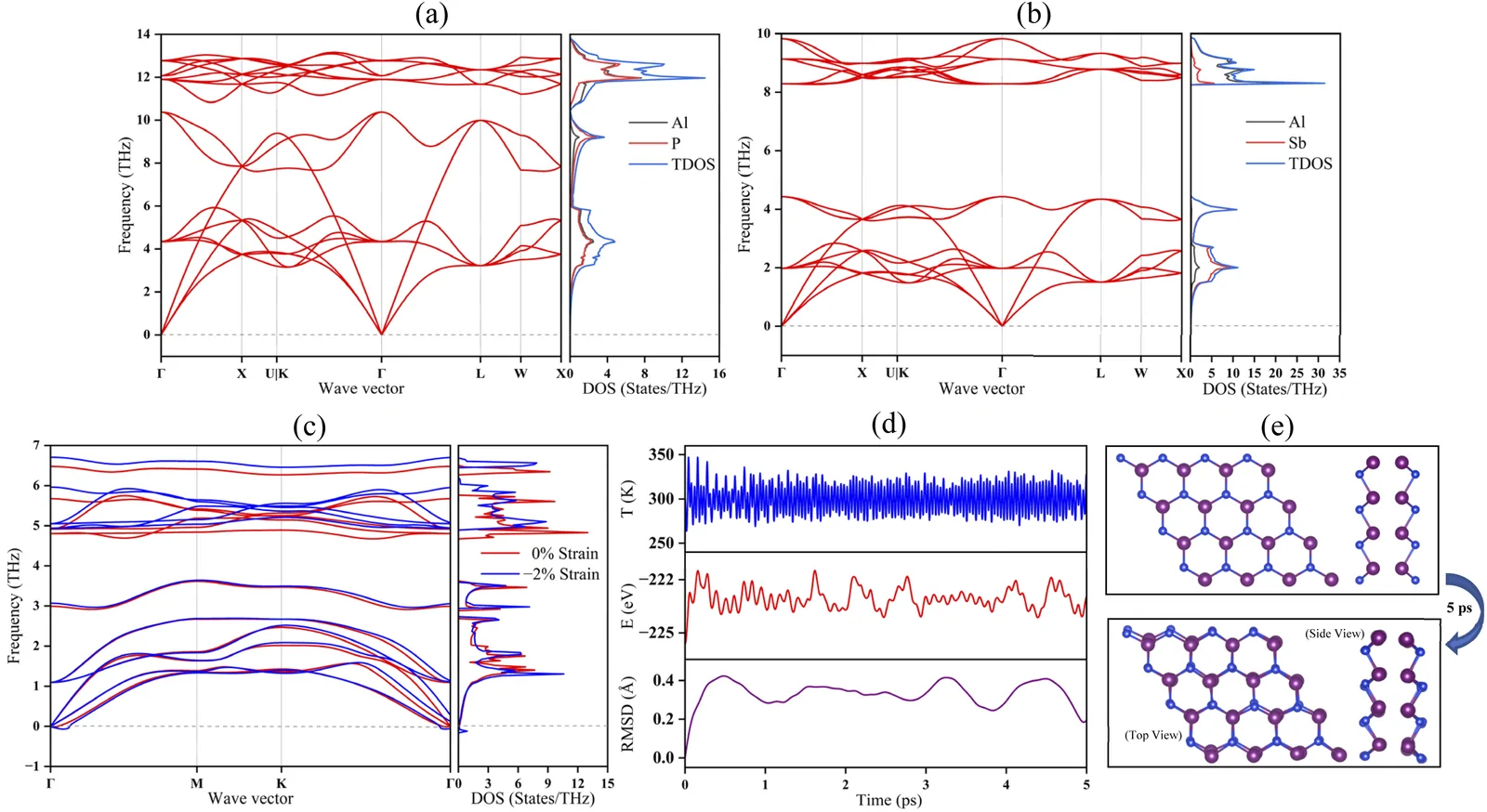
| Phonon dispersions and phonon DOS for (a and b) unstrained bulk AlP, AlSb and (c) InSe monolayer at 0% (red) and −2% (blue) biaxial strain. (d) Time evolution of total energy, temperature, and RMSD from AIMD of InSe monolayer at −2% strain and 300 K. (e) Representative initial and final atomic structures of InSe after 5 ps of molecular dynamics simulation.

To further examine the strain-dependent structural stability, phonon dispersions and phonon densities of states (DOS) were analyzed for all investigated strain states. SI Fig. S1 and S2 show that both AlP and AlSb maintain positive phonon frequencies throughout the Brillouin zone across the entire strain range, confirming robust dynamical stability together with clearly separated acoustic and optical phonon branches. In the phonon DOS, AlP exhibits broadly mixed contributions from Al and P throughout the frequency range because of their relatively comparable atomic masses, whereas AlSb shows a distinct mass-dependent separation, with Al dominating the high-frequency optical region and Sb contributing primarily to the low-frequency acoustic modes, as illustrated in Fig. 2a and b.

In contrast, monolayer InSe remains dynamically stable at equilibrium and under tensile strain but develops small imaginary phonon frequencies localized near the *Γ* point under compressive biaxial strain, as shown in Fig. 2c. Comparable strain-induced *Γ*-point softening has also been reported in previous studies on two-dimensional antimonene, where compressive strain induces imaginary phonon frequencies.^51^ The magnitudes of these soft modes are approximately −0.065, −0.126, and −0.170 THz at −2%, −4%, and −6% strain, respectively. To evaluate possible finite-size effects, additional phonon calculations were performed using larger 5 × 5 × 1 supercells for the compressively strained configurations. SI Fig. S3 demonstrates that the *Γ*-point soft mode persists and slightly increases in magnitude relative to the 4 × 4 × 1 results, indicating that the observed softening is intrinsic within the harmonic approximation rather than arising from numerical artifacts.

Finite-temperature stability was further examined through AIMD simulations at 300 K. Representative AIMD results for compressively strained InSe shown in Fig. 2d exhibit bounded atomic RMSD values of approximately 0.25–0.40 Å, stable total-energy evolution with only small fluctuations, and temperature distributions centred around 300 K throughout the 5 ps simulation.

SI Fig. S4 and S5 further demonstrate stable AIMD behaviour for AlP and AlSb across all investigated strain states without any irreversible structural distortion. Although AlP displays relatively larger instantaneous temperature fluctuations, the average temperature remains close to 300 K throughout the simulation. SI Fig. S6 likewise shows stable RMSD and energy profiles for monolayer InSe under all strain states, indicating the absence of immediate structural collapse within the accessible picosecond timescale.

Overall, the combined structural, phonon, and AIMD analyses demonstrate that AlP and AlSb remain dynamically and thermally stable throughout the investigated biaxial strain range, despite the strain-induced symmetry reduction from cubic to tetragonal phases. Monolayer InSe develops shallow harmonic soft modes under compressive strain; however, the absence of significant structural reconstruction or energy divergence during AIMD simulations indicates that the strained monolayer retains finite-temperature stability at 300 K within the simulated timescale. This behaviour indicates that the observed instability remains weak within the harmonic approximation and does not immediately induce structural collapse at finite temperature. These results further demonstrate that the choice of exchange–correlation functional substantially influences the structural and energetic reference states, which consequently affect subsequent predictions of mechanical, electronic, and optical properties.

### 3.2 Thermodynamic properties

**Fig. 3:**
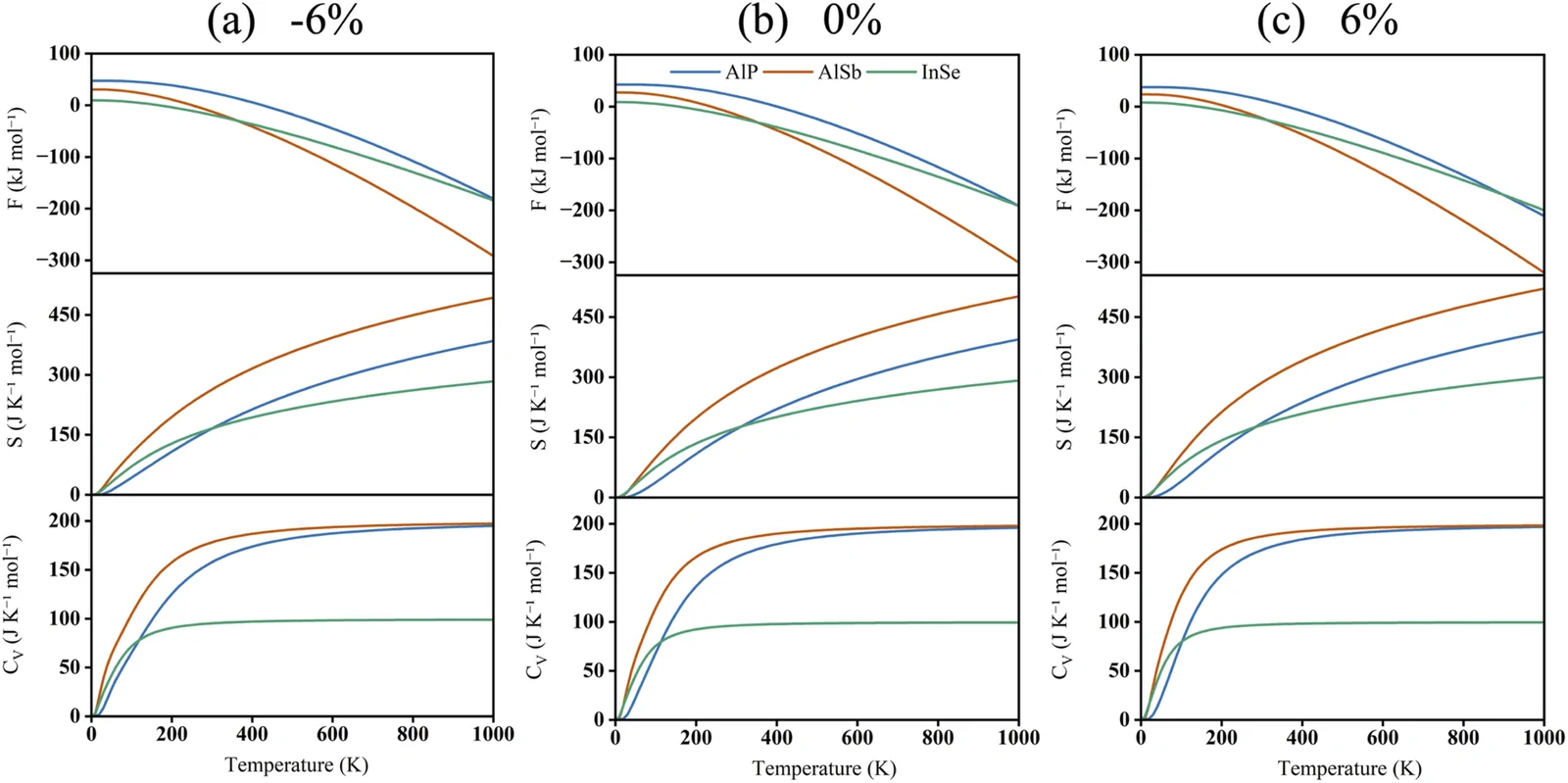
| Temperature-dependent thermodynamic properties of bulk AlP, bulk AlSb, and monolayer InSe under representative biaxial strain states. Panels (a–c) correspond to −6%, 0%, and +6% biaxial strain, respectively. Within each panel, the Helmholtz free energy (*F*), entropy (*S*), and constant–volume heat capacity (*C*_V_) are shown as functions of temperature. The thermodynamic properties were calculated from the phonon spectra within the harmonic approximation.

To further examine the effect of biaxial strain on lattice dynamics, the temperature-dependent thermodynamic properties of bulk AlP, bulk AlSb, and monolayer InSe were evaluated within the harmonic approximation using the phonon spectra. The Helmholtz free energy (*F*), entropy (*S*), and constant–volume heat capacity (*C*_V_) were calculated over the temperature range of 0–1000 K for representative compressive (−6%), equilibrium (0%), and tensile (+6%) strain states, as shown in Fig. 3.

Fig. 3(a–c) show that the Helmholtz free energy decreases monotonically with increasing temperature for all three materials, irrespective of the applied strain. This behaviour arises from the increasing vibrational entropy contribution to the lattice free energy. As the temperature increases, more phonon modes become thermally populated, leading to an increase in vibrational entropy and causing the −*TS* term in the Helmholtz free energy expression (*F* = *U* − *TS*) to become increasingly dominant. Consequently, the free energy decreases continuously with temperature. Although slight quantitative differences are observed between compressive and tensile strain states, the overall temperature dependence remains essentially unchanged, indicating that moderate biaxial strain has only a limited influence on the vibrational free energy.

In contrast, the entropy increases continuously with temperature for all investigated systems. At low temperatures, only low-energy phonon modes are appreciably occupied, resulting in relatively small entropy values. With increasing temperature, additional phonon modes become thermally accessible, increasing the number of vibrational microstates and, consequently, the entropy. Among the investigated materials, bulk AlSb exhibits the highest entropy throughout the studied temperature range, whereas monolayer InSe shows the lowest values. These differences reflect the distinct phonon spectra and lattice dynamics of the three materials.

The constant-volume heat capacity increases rapidly at low temperatures before gradually approaching saturation at elevated temperatures. The initial increase is attributed to the progressive excitation of low-frequency phonon modes, while the high-temperature saturation reflects the near-complete population of thermally accessible vibrational states. At this stage, additional thermal energy primarily increases the energy of already occupied phonon modes rather than activating new ones, causing the heat capacity to approach the classical Dulong–Petit limit. Consistent with the behaviour of the Helmholtz free energy and entropy, the influence of biaxial strain on the heat capacity is relatively small, suggesting that moderate lattice deformation does not significantly alter the phonon population or the overall lattice vibrational response.

Overall, the calculated thermodynamic properties indicate that bulk AlP, bulk AlSb, and monolayer InSe exhibit similar temperature-dependent behaviour under both compressive and tensile biaxial strain. The persistence of the characteristic trends in *F*, *S*, and *C*_V_ demonstrates that the vibrational thermodynamic response of these materials remains robust against moderate strain, consistent with their dynamically stable phonon spectra. The calculated temperature dependence of the entropy and constant-volume heat capacity is in good agreement with the quasi-harmonic first-principles study of bulk AlP by Ehsanfar *et al.*,^52^ who likewise reported a monotonic increase in both quantities with increasing temperature. Similarly, the observed decrease in Helmholtz free energy together with the increase in entropy and heat capacity is consistent with recent first-principles thermodynamic calculations on bulk AlSb,^53^ further supporting the reliability of the present phonon-based thermodynamic analysis.

### 3.3 Mechanical properties and elastic anisotropy

**Fig. 4:**
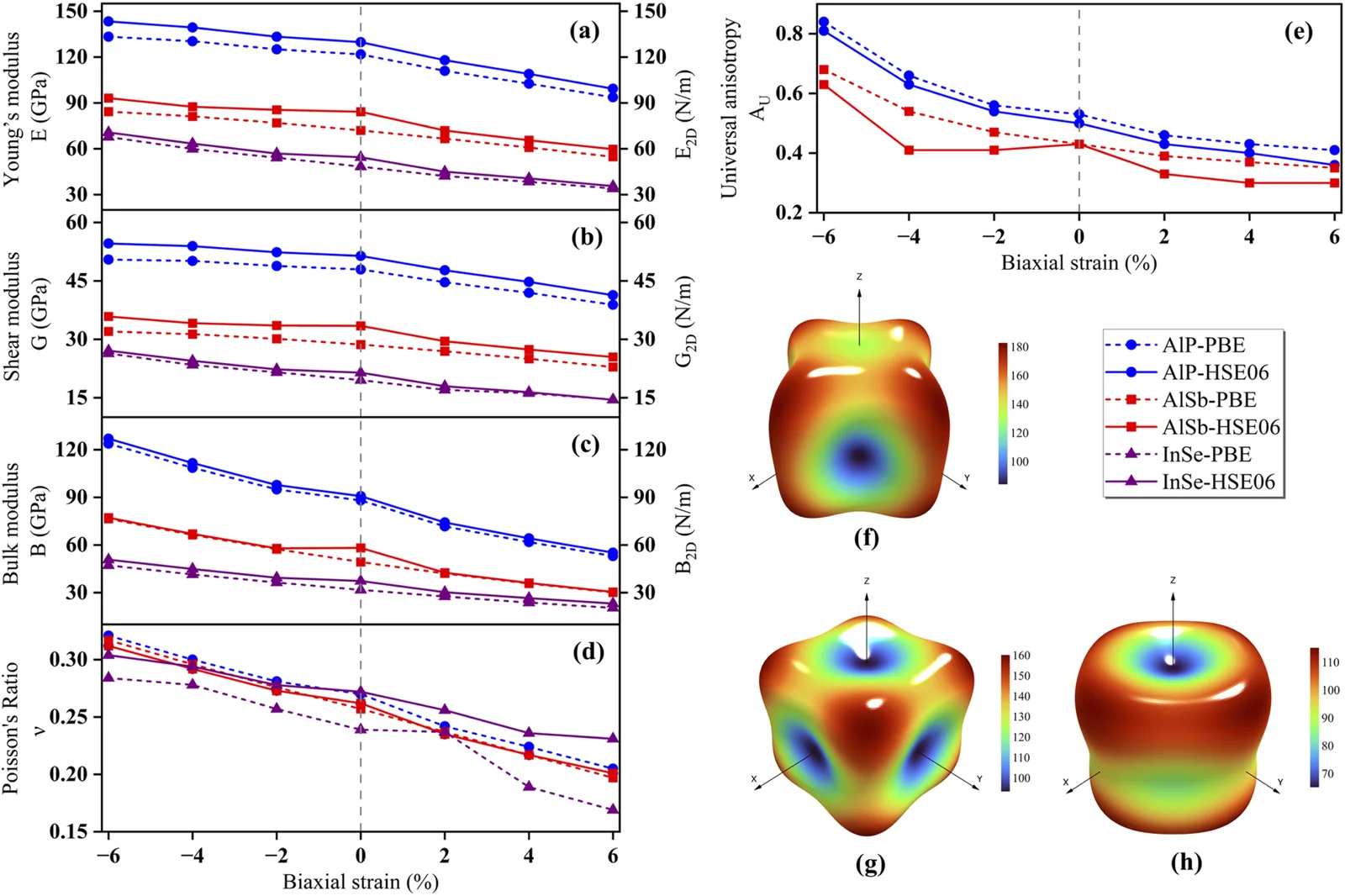
| Strain-dependent elastic response and anisotropy. (a–d) Young's modulus (*E*), shear modulus (*G*), bulk modulus (*B*) and Poisson's ratio (*ν*) of bulk AlP, bulk AlSb and monolayer InSe as functions of biaxial strain, computed with PBE and HSE06. Left and right *y*-axes in (a–c) show three-dimensional (GPa) and in-plane 2D (N m^−1^) units, respectively. (e) Universal elastic anisotropy index (*A*_U_) for bulk AlP and AlSb. (f–h) Directional Young's modulus surfaces of bulk AlP (HSE06) at −6%, 0% and +6% biaxial strain, illustrating the evolution of elastic anisotropy following strain-induced symmetry lowering.

The strain-dependent mechanical response of bulk AlP, bulk AlSb, and monolayer InSe was systematically investigated using the PBE and HSE06 functionals under biaxial strain ranging from −6% to +6%. Full elastic tensors and strain-resolved elastic moduli are provided in SI Tables S2 and S3, whereas the main text focuses on the overall trends and their physical interpretation based on Fig. 4. All strained structures satisfy the Born mechanical stability criteria throughout the investigated biaxial strain range (−6% to +6%), confirming their elastic stability (Table S2).

Fig. 4(a–d) summarize the evolution of Young's modulus (*E*), shear modulus (*G*), bulk modulus (*B*), and Poisson's ratio (*ν*) with strain. For both bulk AlP and AlSb, *E*, *G*, and *B* increase monotonically under compressive strain and decrease under tensile strain, reflecting progressive bond strengthening and weakening, respectively. At equilibrium, AlP exhibits consistently larger elastic moduli than AlSb, indicating a stiffer lattice. Under tensile strain, AlSb shows a stronger reduction in stiffness than AlP, consistent with its comparatively softer bonding character. Across the entire strain range, HSE06 predicts systematically higher elastic moduli than PBE (typically by 6–12%), owing to the more accurate treatment of exchange interactions within the hybrid functional, while both functionals capture identical qualitative strain dependence. Poisson's ratio decreases smoothly from compressive to tensile strain for both bulk materials, indicating enhanced lateral contraction under compression and reduced transverse response under tension. The strain dependence of *ν* is slightly weaker in AlSb than in AlP, consistent with the softer elastic response of AlSb.

For monolayer InSe, the in-plane elastic moduli (*E*_2D_, *G*_2D_, and *B*_2D_) display qualitatively similar strain trends but markedly higher strain sensitivity compared to the bulk compounds. Tensile strain leads to a substantial reduction of in-plane stiffness, while compressive strain enhances rigidity, reflecting the intrinsic mechanical flexibility of 2D systems. HSE06 again predicts systematically larger elastic moduli than PBE, while preserving the same qualitative strain dependence (Table S3). Poisson's ratio of InSe varies only modestly with strain, indicating a stable in-plane deformation mechanism.

The strain-induced evolution of elastic anisotropy is quantified using the universal elastic anisotropy index *A*_U_, shown in Fig. 4e for bulk AlP and AlSb. At equilibrium, both materials exhibit finite elastic anisotropy, which increases under compressive strain and decreases under tensile strain. AlP displays a stronger anisotropic response than AlSb over the full strain range. For both compounds, HSE06 predicts systematically lower *A*_U_ values than PBE, indicating a more uniform elastic response when hybrid exchange is included. These trends correlate with the strain-induced symmetry reduction from the cubic zinc blende structure (*F*4̄3*m*) to tetragonal symmetry (*I*4̄*m*2), which lifts the elastic degeneracies characteristic of the cubic phase and introduces direction-dependent stiffness. In the unstrained zinc blende structure, the nearest-neighbour bonds are related by cubic symmetry, resulting in equivalent elastic responses along symmetry-equivalent directions. Under biaxial strain, the in-plane and out-of-plane lattice responses become inequivalent, leading to anisotropic changes in bond lengths and bond angles. This strain-induced redistribution of bonding environments modifies the directional elastic response, as reflected in the calculated elastic tensor and Young's modulus surfaces, and consequently produces changes in the universal elastic anisotropy index, *A*_U_. Compressive strain enhances the contrast between different crystallographic directions, whereas tensile strain reduces the elastic anisotropy in the present systems. Fig. 4(f–h) present the directional Young's modulus surfaces of bulk AlP calculated using HSE06 at −6%, 0%, and +6% strain. At equilibrium, Young's modulus exhibits pronounced directional dependence, which is further modified under both compressive and tensile strain due to symmetry lowering. Compressive strain enhances elastic contrast among crystallographic directions, whereas tensile strain redistributes stiffness and reduces the overall magnitude of directional moduli. Equivalent geometric anisotropy patterns are obtained using PBE, confirming that both functionals capture the same anisotropy topology despite moderate quantitative differences. Complete directional anisotropy plots for Young's modulus, shear modulus, bulk modulus, and Poisson's ratio of AlP and AlSb at representative strain values are provided in SI Fig. S7 and S8. In contrast, monolayer InSe remains elastically isotropic within the in-plane directions for all applied strains and for both functionals, resulting in a vanishing anisotropy index (*A*_U_ ≈ 0). Consequently, directional elastic surfaces are not presented for monolayer InSe.

### 3.4 Electronic structure and band evolution

The electronic structure was systematically benchmarked using three exchange–correlation treatments to establish quantitative accuracy and strain tunability. Calculations were performed with the PBE-GGA and the HSE06 hybrid functional employing two exact-exchange mixing ratios (25% and 30%), enabling an assessment of functional reliability across bulk semiconductors and 2D systems while balancing computational cost and accuracy.

**Fig. 5:**
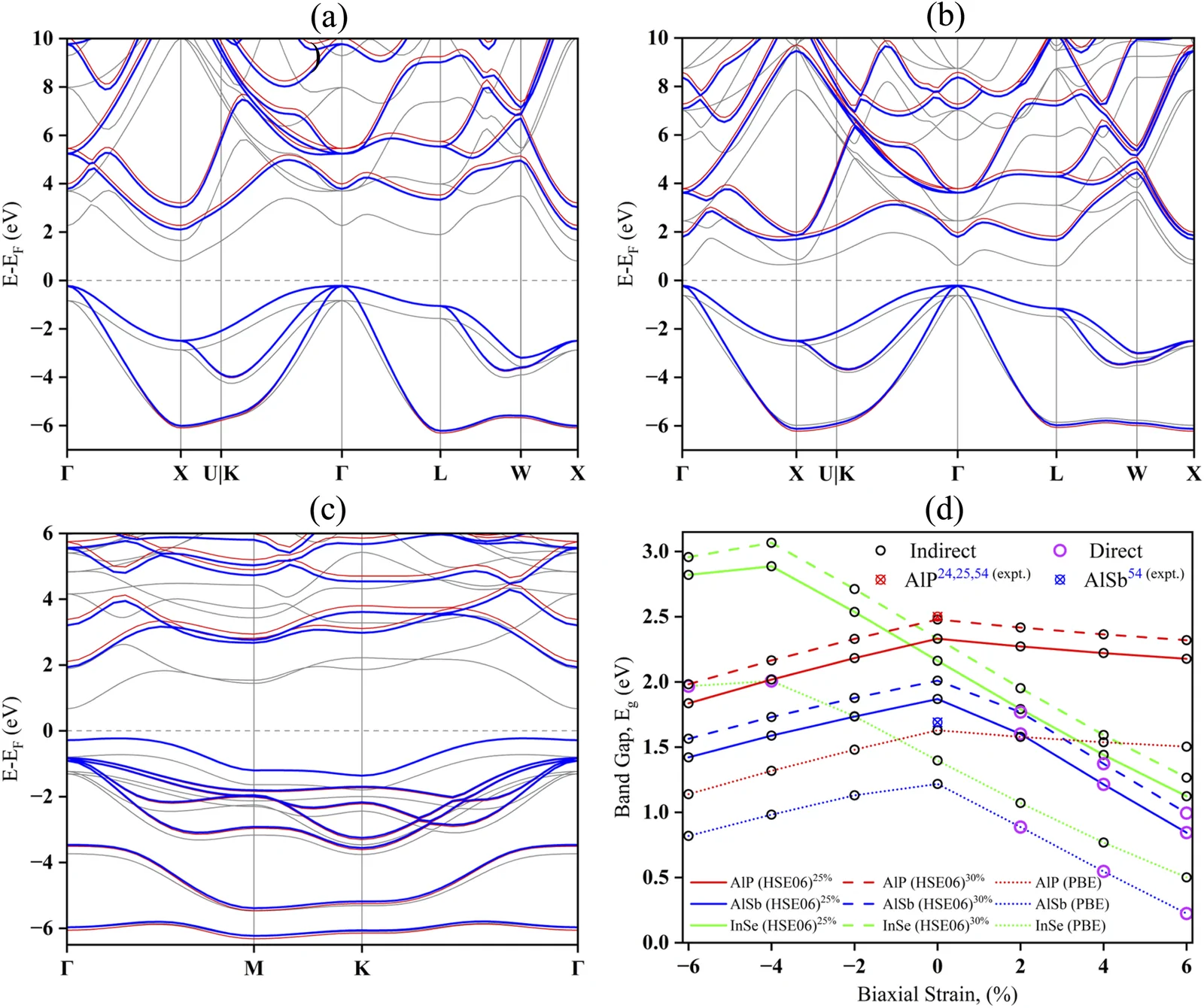
| Electronic band structures and strain-dependent band gaps of bulk AlP, bulk AlSb, and monolayer InSe. Panels (a–c) show the band structures of the unstrained systems calculated using PBE (gray), HSE06-25% (blue), and HSE06-30% (red). Panel (d) presents the evolution of the band gap as a function of biaxial strain for all three compounds; symbols distinguish indirect and direct gaps as indicated in the legend.

At zero strain, HSE06 with 30% exact exchange (HSE06-30%) yields an indirect band gap of 2.48 eV for bulk AlP, in excellent agreement with the experimental value of 2.50 eV, whereas PBE significantly underestimates the gap at 1.63 eV (Fig. 5a).^54^ For monolayer InSe, HSE06-30% predicts a band gap of 2.33 eV, substantially larger than the PBE value of 1.40 eV and consistent with the layer-dependent band gap increase reported for few-layer InSe from photoluminescence measurements (Fig. 5b).^55,56^ In contrast, bulk AlSb is better described by HSE06 with 25% exact exchange (HSE06-25%), which yields a gap of 1.87 eV, close to the experimental indirect gap of 1.69 eV, while HSE06-30% predicts 2.01 eV and PBE yields only 1.22 eV (Fig. 5c).^54^ The HSE06-25% variant systematically reduces the band gap by approximately 0.15 eV relative to the 30% mixing while preserving all strain-dependent trends, indicating that the exact-exchange fraction primarily scales the band-gap magnitude without altering qualitative behaviour.

**Fig. 6:**
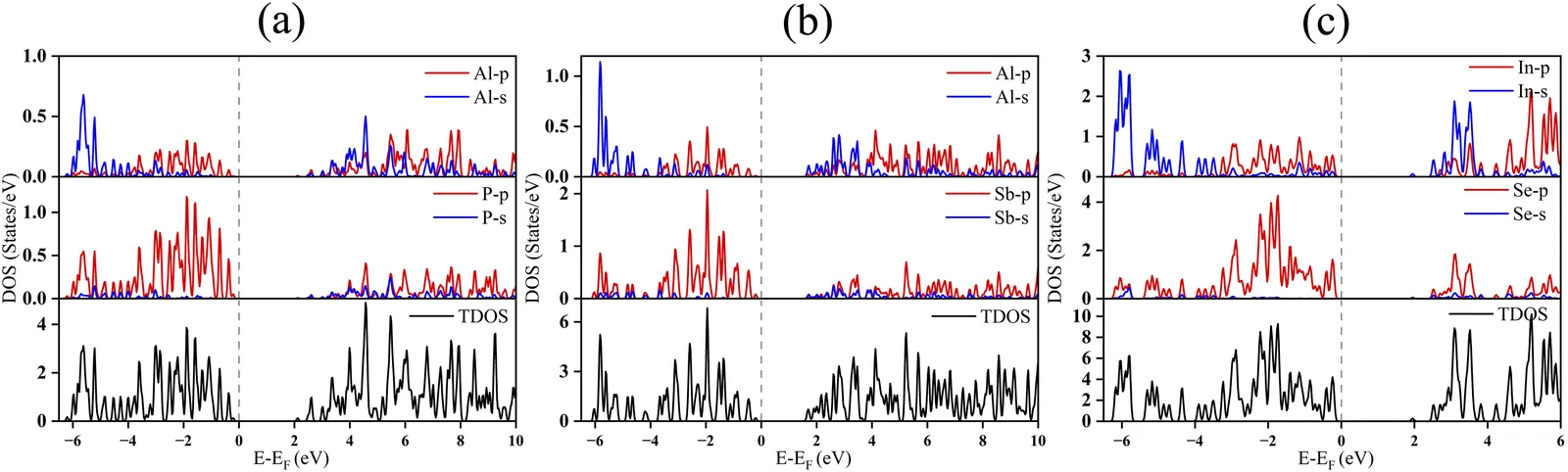
| Projected density of states (PDOS) of the equilibrium (0% strain) structures of (a) bulk AlP, (b) bulk AlSb, and (c) monolayer InSe calculated using the HSE06 hybrid functional. The Fermi level is referenced to 0 eV.

The magnitude of the hybrid-functional correction is notably larger for monolayer InSe (0.93 eV difference between HSE06-30% and PBE at zero strain) than for the bulk compounds (0.85 eV for AlP and 0.79 eV for AlSb), reflecting reduced dielectric screening and enhanced sensitivity to exchange effects in low-dimensional systems. As shown in Fig. 6, the equilibrium HSE06 projected density of states (PDOS) reveals the orbital characteristics of the electronic states near the band edges. For bulk AlP, the valence band is dominated by P-p states with a minor contribution from Al-p states, whereas the conduction band consists of hybridized Al-s, Al-p, P-p, and P-s states, indicating significant orbital hybridization. Bulk AlSb shows a similar valence-band composition, where Sb-p states dominate with a small contribution from Al-p states, while the conduction band is primarily derived from Al-s states with weaker contributions from the remaining orbitals. In monolayer InSe, the valence band is mainly composed of Se-p states accompanied by a minor In-p contribution, whereas the conduction band is dominated by In-s states together with noticeable Se-p contributions. The strain-dependent evolution of the orbital-resolved PDOS under representative biaxial strains (−6%, 0%, and +6%) is presented in SI Fig. S12–S14.

Complete band structures for all strain states are provided in SI Fig. S9–S11. Under biaxial strain (±6%), the three compounds exhibit distinct electronic responses. Bulk AlP shows a maximum band gap at zero strain, decreasing more rapidly under compressive than tensile strain, with all gaps remaining indirect for all functionals. The total modulation across ±6% strain is approximately 0.50 eV for all functionals (Fig. 5d), indicating moderate strain tunability and robust gap character. In contrast, bulk AlSb undergoes an indirect-to-direct band-gap transition under tensile strain (≥+2%) for all three functionals, arising from strain-induced reordering of conduction-band minima. AlSb exhibits the strongest strain modulation among the bulk systems, with band-gap ranges of 1.03 eV (HSE06-25%) and 1.07 eV (HSE06-30%), showing greater sensitivity to tensile than compressive strain unlike AlP, while preserving the systematic 0.15–0.20 eV offset between mixing ratios across all strain states. The cubic-to-tetragonal symmetry reduction (*F*4̄3*m* → *I*4̄*m*2) in AlP and AlSb under biaxial strain, discussed in the Structural properties section, does not alter the indirect band-gap character in AlP despite lifting degeneracies at *Γ*. In AlSb, however, this symmetry lowering enables direct-indirect competition, and tensile strain causes sufficient downshifting of the *Γ*-point conduction band relative to the *X*-point CBM to make the direct *Γ*–*Γ* gap energetically favourable, while compressive strain maintains indirect character.

Monolayer InSe displays the most pronounced strain sensitivity. Within HSE06-30%, the band gap peaks at ∼3.0 eV at −4% strain before decreasing to 1.27 eV at +6% strain, a total tuning range of 1.69 eV substantially exceeding the bulk compounds. HSE06 predicts an indirect band gap across the full strain range, whereas PBE indicates a direct gap at large compressive strains (−6% and −4%), highlighting the sensitivity of band-edge character in 2D systems to exchange–correlation treatment. The reduced HSE06 to PBE gap ratio for InSe under compression compared with the bulk compounds reflects strain-dependent screening effects unique to low-dimensional materials. The preservation of hexagonal symmetry (P6̄*m*2) throughout the strain range further contributes to the stability of the indirect band-gap character in InSe, in contrast to the symmetry-breaking transitions observed in the bulk compounds.

Overall, HSE06-30% provides the most reliable quantitative description of band gaps for bulk AlP and monolayer InSe, while HSE06-25% yields closer agreement for bulk AlSb. Both hybrid variants reproduce identical strain trends with systematically tuneable gaps. Although PBE underestimates absolute band gaps, it captures functional-independent strain responses and remains suitable for preliminary screening. These results demonstrate moderate strain tunability in bulk AlP and AlSb, exceptional band-gap modulation in monolayer InSe, and the critical interplay between structural symmetry and electronic gap character in determining strain-engineered electronic behaviour.

### 3.5 Optical properties

**Fig. 7:**
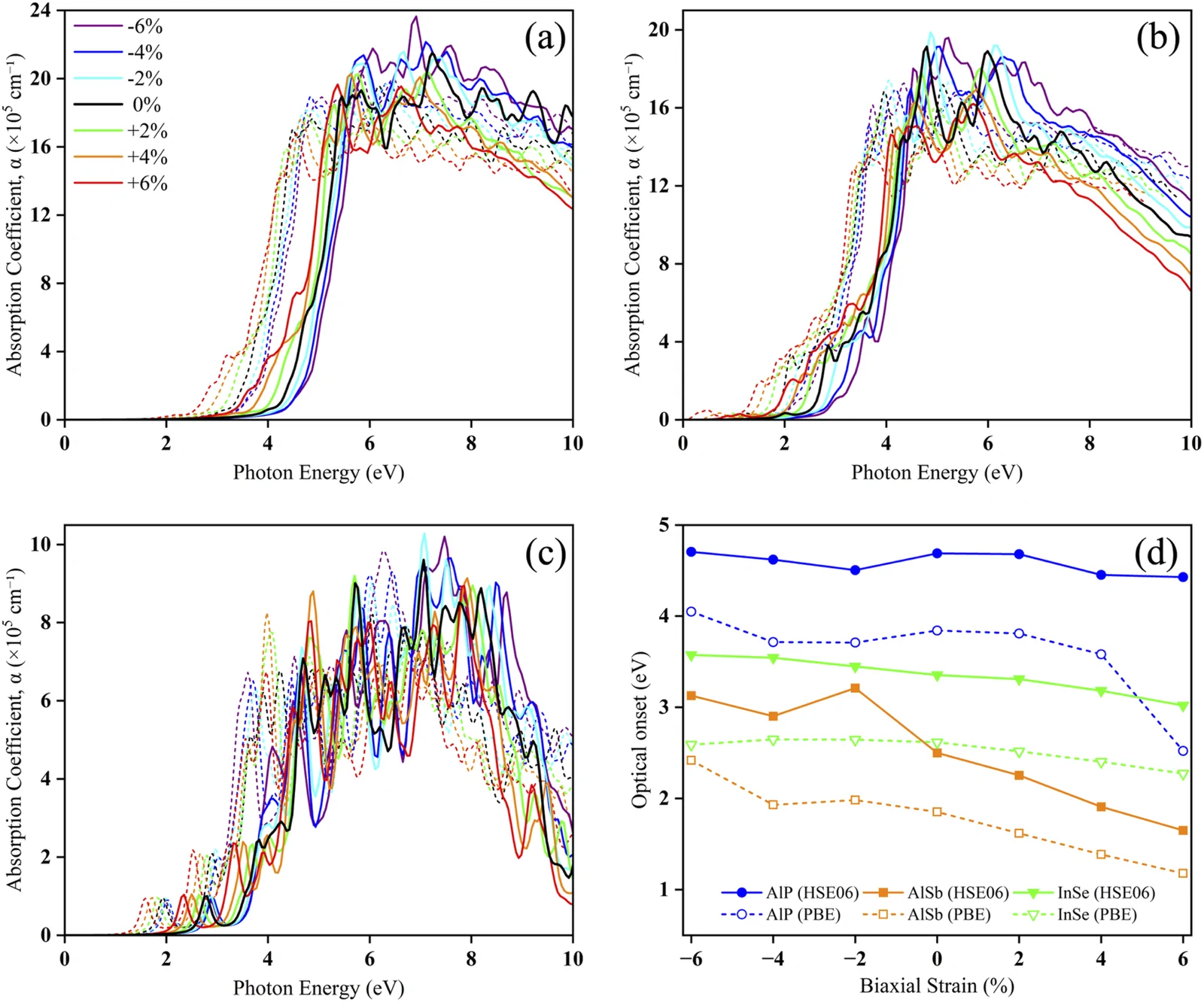
| Optical absorption spectra and strain-dependent optical onset of bulk AlP, bulk AlSb, and monolayer InSe under biaxial strain. Panels (a–c) show the calculated absorption coefficients of (a) AlP, (b) AlSb, and (c) InSe for biaxial strains ranging from −6% to +6%. Panel (d) summarizes the variation of the optical onset with biaxial strain. Solid and dashed lines represent the HSE06 and PBE results, respectively.

The strain-dependent optical properties of bulk AlP, bulk AlSb, and monolayer InSe were investigated using both the PBE and HSE06 exchange–correlation functionals. The calculated absorption spectra are presented in Fig. 7(a–c), while the corresponding optical onset as a function of biaxial strain is summarized in Fig. 7d. The optical onset was extracted automatically from the low-energy absorption edge using an automated linear-fitting procedure, with the detailed algorithm and a representative fitting example provided in SI Fig. S17.

Fig. 7(a–c) demonstrate that the absorption spectra of all three materials exhibit a pronounced dependence on biaxial strain, particularly near the absorption edge. Overall, the optical onset broadly follows the strain-dependent band-gap evolution and generally shifts toward lower photon energies under tensile biaxial strain, whereas compressive strain tends to produce a corresponding blueshift.^57,58^ Compared with PBE, HSE06 predicts a systematic shift of the absorption edge toward higher photon energies, resulting in consistently larger optical onset values. At higher photon energies, however, both functionals produce similar spectral profiles and exhibit nearly identical strain-dependent evolution, indicating that the hybrid functional primarily shifts the excitation threshold while preserving the overall spectral characteristics. The absorption spectra also reveal that bulk AlP exhibits the strongest absorption intensity, followed by bulk AlSb and monolayer InSe over most of the investigated photon-energy range.

The strain dependence of the extracted optical onset is summarized in Fig. 7d. For all three materials, the HSE06 optical onset is consistently larger than the corresponding PBE value, consistent with the larger electronic band separations predicted by the hybrid functional. Among the investigated materials, bulk AlSb exhibits the most pronounced strain dependence, with the HSE06 optical onset decreasing from approximately 3.13 eV at −6% strain to 1.65 eV at +6% strain. In comparison, bulk AlP shows only a modest reduction from 4.71 eV to 4.43 eV, whereas monolayer InSe exhibits an intermediate response, decreasing from 3.57 eV to 3.02 eV over the same strain range. The corresponding PBE optical onset follows the same general strain dependence but systematically yields smaller values because of the well-known underestimation of the electronic band gap by semilocal density functionals.

The evolution of the optical onset is closely correlated with the strain-dependent electronic structure discussed in the previous section. Since optical absorption predominantly arises from momentum-conserving interband transitions, the optical onset is expected to be associated with the lowest optically allowed transition rather than the fundamental indirect band gap.^59,60^ The quantitative distinction between the fundamental band gap and optical onset is further illustrated in Table 2 at −6%, 0%, and +6% strain. At zero strain, *E*_onset_ − *E*_g_ (eV) is 2.36, 0.63, and 1.19 eV for AlP, AlSb, and InSe, respectively. For AlP and InSe, the difference remains substantial across the selected strain states, consistent with their indirect band-gap character throughout the investigated strain range. This distinction arises because the fundamental band gap represents the minimum energy separation between the valence-band maximum and conduction-band minimum, which may occur at different *k*-points, whereas the optical onset is extracted from the absorption edge arising from optically allowed vertical interband transitions in the calculated absorption spectrum. For AlSb, the difference is comparatively smaller and remains within 0.63–0.81 eV from 0% to +6% strain, where the electronic structure indicates an indirect-to-direct band-gap transition. The reduced *E*_onset_ − *E*_g_ under tensile strain is consistent with the emergence of direct band-edge transitions. These results demonstrate that biaxial strain effectively modulates the optical excitation threshold through its influence on the electronic band structure while preserving the overall optical response.

**Table 2:** Comparison of the HSE06 fundamental band gap, optical onset, and their difference at representative biaxial strains

Material	Strain (%)	*E*_g_ (eV)	*E*_onset_ (eV)	*E*_onset_ − *E*_g_ (eV)
AlP	−6	1.836	4.706	2.870
	0	2.332	4.690	2.358
	+6	2.178	4.428	2.250
AlSb	−6	1.421	3.126	1.705
	0	1.869	2.500	0.631
	+6	0.844	1.650	0.806
InSe	−6	2.821	3.572	0.751
	0	2.162	3.352	1.190
	+6	1.123	3.019	1.896

The frequency-dependent dielectric function, reflectivity, and refractive index are presented in SI Fig. S15 and S16. The real and imaginary parts of the dielectric function exhibit systematic energy shifts that closely follow the evolution of the absorption edge under biaxial strain while maintaining similar spectral features for both exchange–correlation functionals. Likewise, the reflectivity and refractive index display only moderate strain-induced variations throughout the investigated photon-energy range, indicating that biaxial strain primarily modifies the energies of optical transitions rather than the intrinsic optical characteristics of the materials. Collectively, these results demonstrate that biaxial strain provides an effective strategy for tuning the optical properties of bulk AlP, bulk AlSb, and monolayer InSe over a broad spectral range.

## 4 Conclusions

This work presents a unified first-principles assessment of strain-engineered III–V semiconductors (bulk AlP and AlSb) and monolayer InSe, with explicit benchmarking of PBE against HSE06. HSE06 substantially improves structural accuracy, reducing the mean absolute deviation in lattice parameters from 1.47% for PBE to 0.39%, while yielding smaller equilibrium volumes and more exothermic formation energies. These results demonstrate that the choice of exchange–correlation functional strongly influences the structural reference state used to evaluate strain effects.

All materials remain dynamically stable across the ±6% biaxial strain range, as confirmed by phonon spectra and AIMD simulations at 300 K. Phonon analysis combined with short-time AIMD distinguishes harmonic softening from genuine finite-temperature instability. Bulk AlP and AlSb exhibit no imaginary phonon modes and remain stable in AIMD despite symmetry lowering from cubic zinc blende to tetragonal structures. Monolayer InSe develops shallow *Γ*-point soft modes under compressive strain, but bounded atomic displacements and stable total energies confirm its room-temperature stability.

Thermodynamic calculations further show that the temperature dependence of the Helmholtz free energy, entropy, and constant-volume heat capacity remains largely insensitive to moderate biaxial strain, demonstrating that the vibrational thermodynamic behaviour of all three materials is robust across the investigated strain range.

Mechanically, biaxial strain lowers the crystal symmetry of bulk AlP and AlSb from cubic to tetragonal, while compressive strain enhances elastic anisotropy and tensile strain reduces it, with AlP exhibiting the strongest anisotropic response. In contrast, monolayer InSe remains isotropic in-plane, reflecting its intrinsic two-dimensional flexibility. HSE06 predicts slightly higher elastic moduli and marginally lower anisotropy than PBE while preserving identical strain trends.

Electronically, hybrid functionals are essential for quantitatively describing the band gaps of these materials. HSE06 with 30% exact exchange reproduces the experimental band gaps of AlP and InSe, whereas 25% is optimal for AlSb. Under biaxial strain, AlP exhibits moderate indirect-gap tuning, AlSb undergoes a tensile-driven indirect-to-direct band-gap transition, and InSe shows the largest band-gap tunability (approximately 1.69 eV from −6% to +6% strain) while remaining an indirect semiconductor. The optical response closely follows these electronic trends, with tensile strain generally shifting the absorption edge toward lower photon energies, while HSE06 consistently predicts larger optical onsets than PBE without altering the overall strain dependence of the spectra.

Overall, these results establish a robust hybrid-functional framework for understanding the strain-dependent structural, mechanical, thermodynamic, electronic, and optical properties of bulk and two-dimensional III–V semiconductors. The demonstrated combination of structural stability, broad strain tunability, and controllable optoelectronic response highlights the potential of these materials for flexible electronics, strain-engineered optoelectronic devices, photonic technologies, and next-generation sensors and transistors.

## Author contributions

M. T. A.: conceptualization, methodology, software, formal analysis, investigation, data curation, visualization, writing – original draft, writing – review & editing. M. S. A.: supervision, project administration, writing – review & editing. M. S., M. S. I., M. R. S., M. H. R., M. A. A., R. P., and S. H. N.: writing – review & editing. All authors reviewed and approved the final version of the manuscript and agreed to be accountable for all aspects of the work.

## Conflicts of interest

The authors declare no competing interests.

## Supplementary Material

RA-OLF-D6RA06960F-s001

## Data Availability

The data that support the findings of this study are available from the corresponding author upon reasonable request. Supplementary information (SI): strain-dependent structural parameters, phonon dispersion spectra, AIMD results, elastic constants and elastic anisotropy analysis, electronic band structures, density-of-states data, and optical properties for all strain states. See DOI: https://doi.org/10.1039/d6ra06960f.
