# Supplementary material for: Hybrid density functional study of strain-engineered III–V semiconductors and monolayer InSe
Source: RSC Adv. 2026 Sep 14;16(44):55013–25. doi: 10.1039/d6ra06960f (PMC13573631; doi:10.1039/d6ra06960f)
Supplement: Supplementary file 1 [file RA-016-D6RA06960F-s001.pdf]

Supplementary Information (SI) for RSC Advances.
This journal is © The Royal Society of Chemistry 2026

Supplementary Information For

Hybrid Density Functional Study of Strain-Engineered III–V Semiconductors and Monolayer InSe

Md. Tanvir Ahmed[1,2], Maahi Sabah[1,2], Md. Saiful Islam[1,2], Mahabuba Rahman Shanta[1,2], Md. Hafijur Rahman[1], Md. Ashraf Ali[4], Ruma Parvin[3], Saleh Hasan Naqib[3], Md. Shahajan Ali[1,2,*]

[1]Department of Physics, Pabna University of Science and Technology, Pabna-6600, Bangladesh.
[2]Theoretical and Experimental Advanced Materials Science Lab (TEAMS), Pabna University of Science and Technology, Pabna-6600, Bangladesh.
[3]Department of Physics, University of Rajshahi, Rajshahi-6205, Bangladesh.
[4]Department of Physics, Chittagong University of Engineering and Technology, Chattogram-4349, Bangladesh.

*Corresponding author email: msali@pust.ac.bd

Table S1 | Structural parameters, bond lengths (*d*), volumes (V), and space groups of bulk AlP, AlSb, and monolayer InSe under biaxial strains (±2%, ±4%, ±6%), calculated using the PBE functional. HSE06 values for unstrained structures are provided in parentheses.

Compound	***Biaxial Strain (%)***	***a (Å)***	***c (Å)***	***d (Å)***	***Space Group***	***V (Å³/f.u.)***
**AlP**	0	5.506 (5.473)	5.506 (5.473)	2.384 (2.370)	$F\bar{4}3m$ (216)	41.735 (40.983)
	-6%	5.176		2.290	$I\bar{4}m2$ (119)	36.877
	-4%	5.286		2.321		38.463
	-2%	5.396		2.353		40.082
	2%	5.616		2.416		43.421
	4%	5.726		2.448		45.140
	6%	5.837		2.481		46.893
**AlSb**	0	6.235 (6.149)	6.235 (6.149)	2.699 (2.663)	$F\bar{4}3m$ (216)	60.588 (58.131)
	-6%	5.861		2.593	$I\bar{4}m2$ (119)	53.535
	-4%	5.985		2.628		55.837
	-2%	6.110		2.664		58.188
	2%	6.359		2.736		63.035
	4%	6.484		2.772		65.531
	6%	6.609		2.809		68.076
**InSe**	0	4.082 (4.034)	25.330 (25.033)	2.682 (2.645)	$P\bar{6}m2$ (187)	182.813 (176.415)
	-6%	3.838		2.627		161.533
	-4%	3.919		2.643		168.480
	-2%	4.001		2.662		175.573
	2%	4.164		2.703		190.198
	4%	4.246		2.724		197.730
	6%	4.328		2.747		205.408

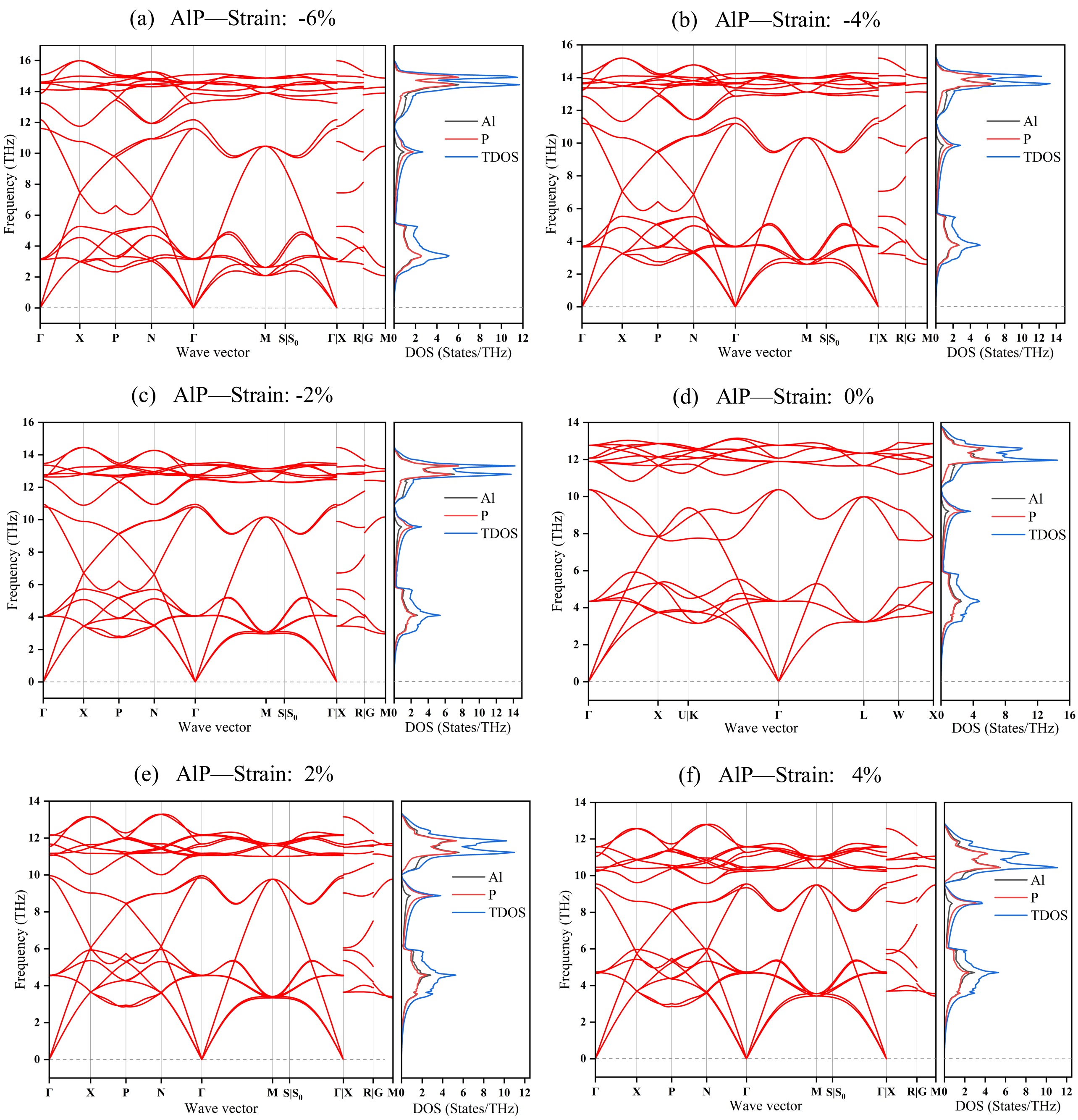

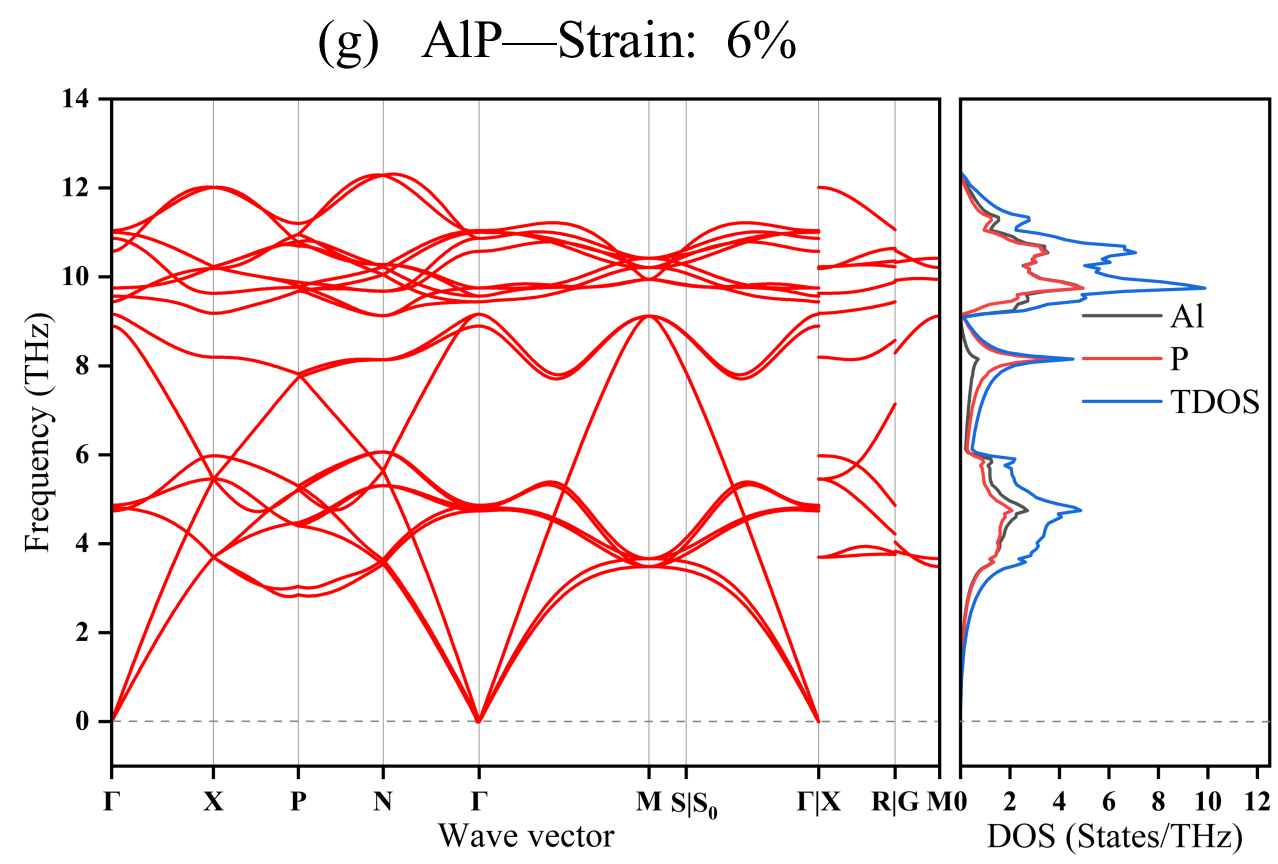

Fig. S1 | Phonon dispersion relations and the corresponding density of states of AlP under various biaxial strain levels. Panels (a)–(g) correspond to strain values of −6%, −4%, −2%, 0%, +2%, +4%, and +6%, respectively.

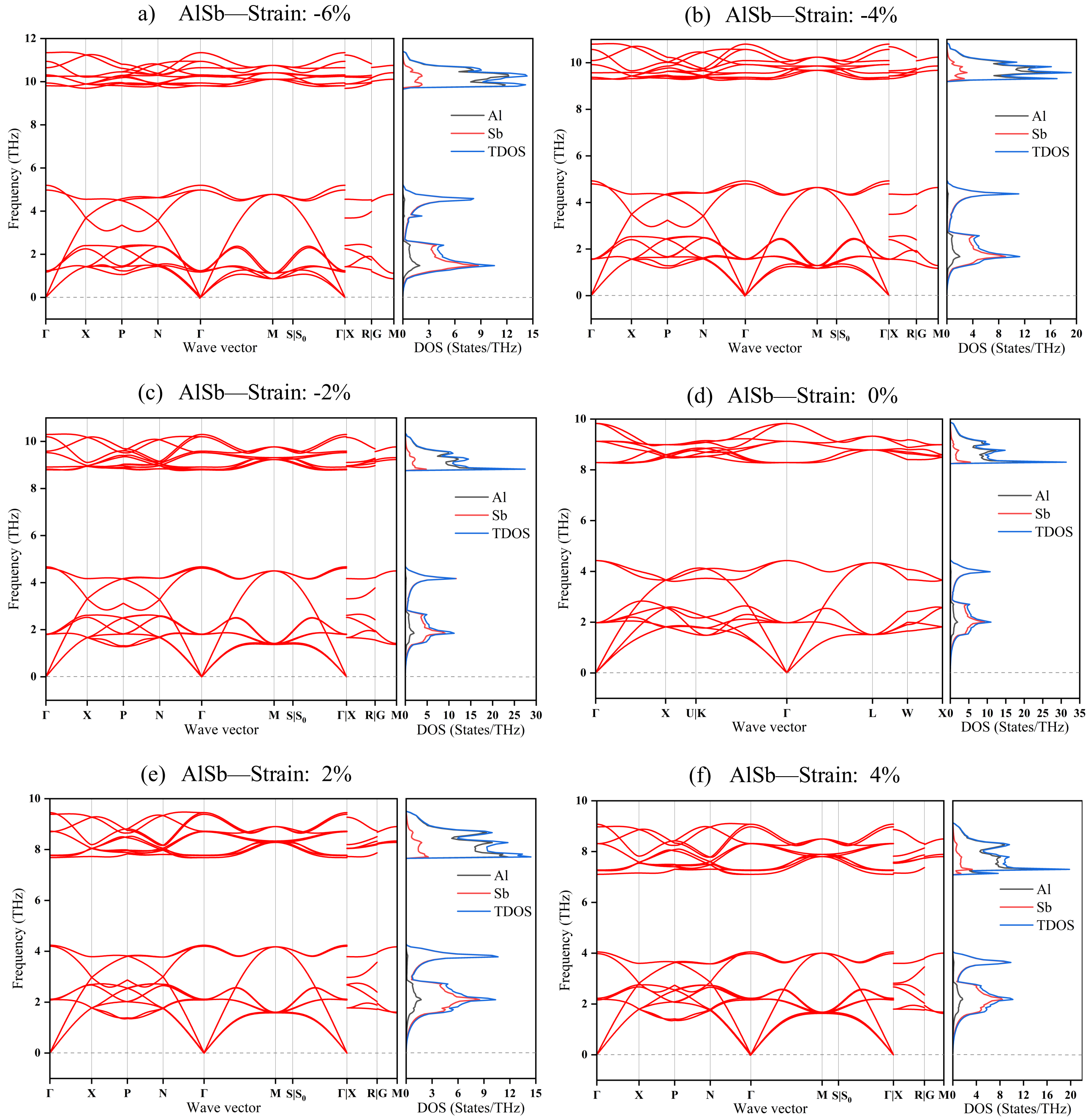

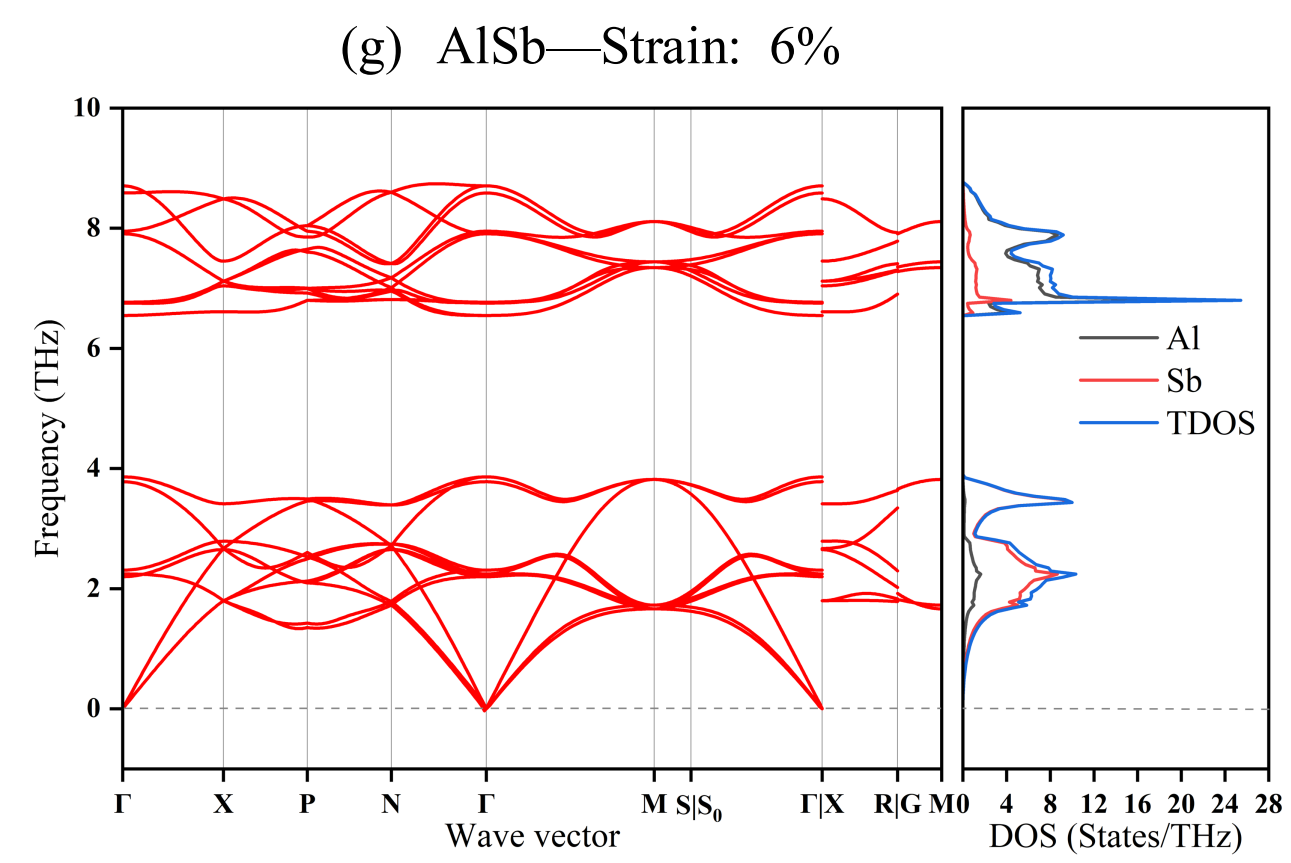

Fig. S2 | Phonon dispersion relations and the corresponding density of states of AlSb under various biaxial strain levels. Panels (a)–(g) correspond to strain values of −6%, −4%, −2%, 0%, +2%, +4%, and +6%, respectively.

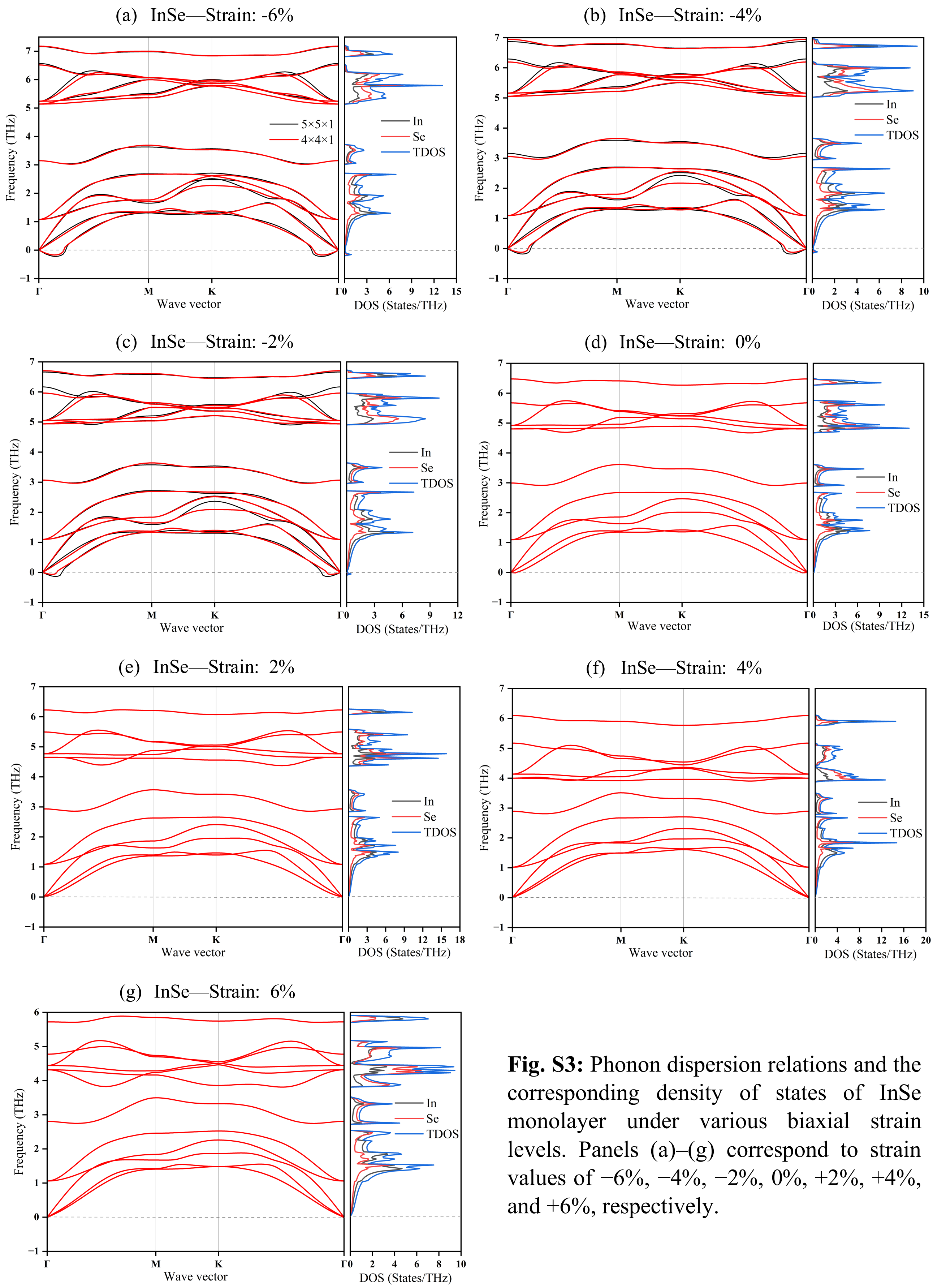

Fig. S3: Phonon dispersion relations and the corresponding density of states of InSe monolayer under various biaxial strain levels. Panels (a)–(g) correspond to strain values of −6%, −4%, −2%, 0%, +2%, +4%, and +6%, respectively.

Fig. S3 shows the phonon dispersions of monolayer InSe under compressive biaxial strain; panels (a–c) correspond to −6%, −4%, and −2% strain, respectively. Solid red lines are the results (with atom-projected PDOS displayed to the right), and solid black lines are the 5×5×1 convergence checks. The Γ-point soft mode persists in the larger 5×5×1 supercell and increases slightly in magnitude compared to the 4×4×1 data, indicating that the imaginary frequency is intrinsic (not a finite-supercell artifact). Despite this shallow harmonic softening, finite-temperature AIMD simulations (see main text and Supplementary Fig. S6) at 300 K do not show runaway structural distortion on the picosecond timescale, suggesting the compressed InSe remains metastable at finite temperature.

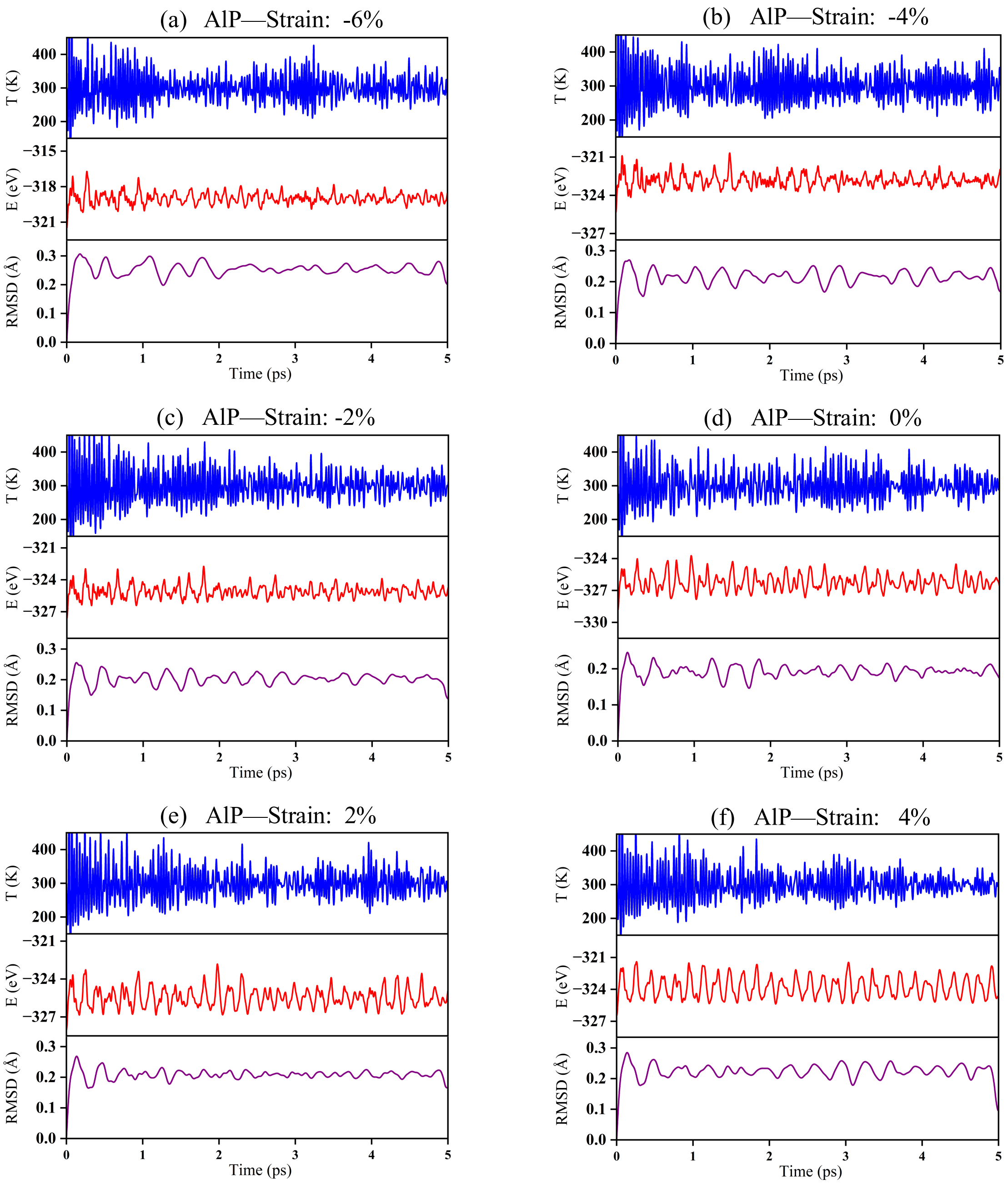

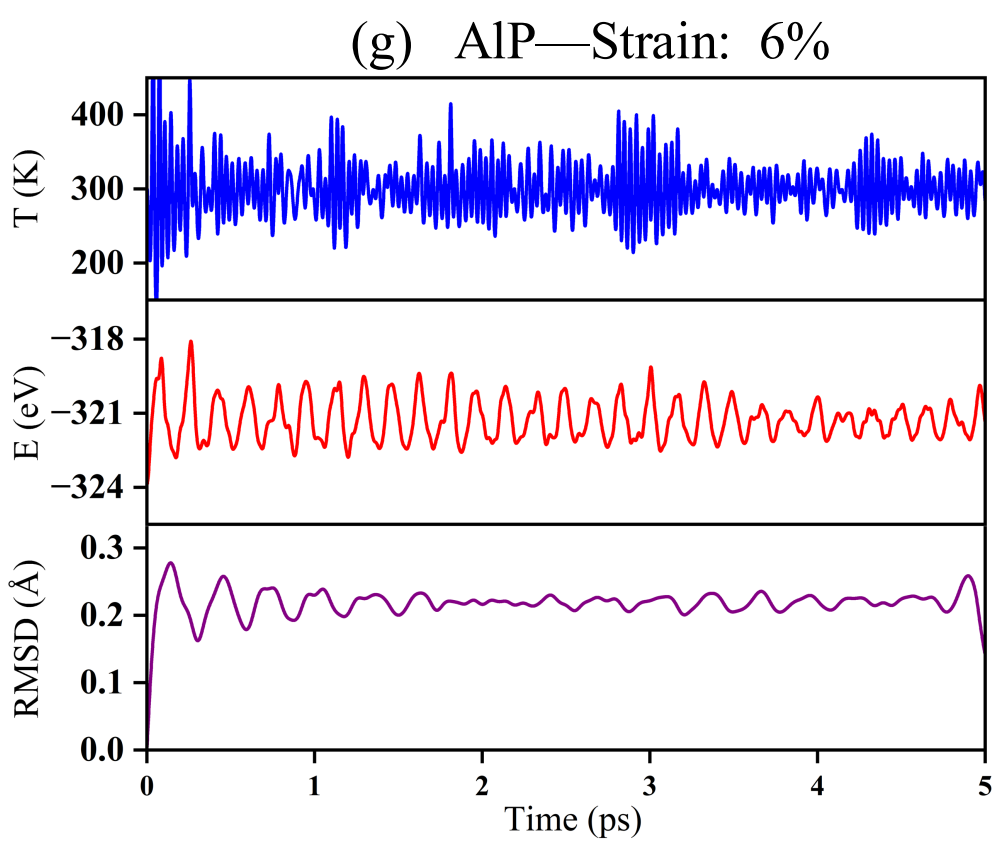

Fig. S4 | Time evolution of temperature, total energy, and RMSD for AlP at 300 K. Panels (a–g) correspond to biaxial strain values of −6%, −4%, −2%, 0%, +2%, +4%, and +6%, respectively.

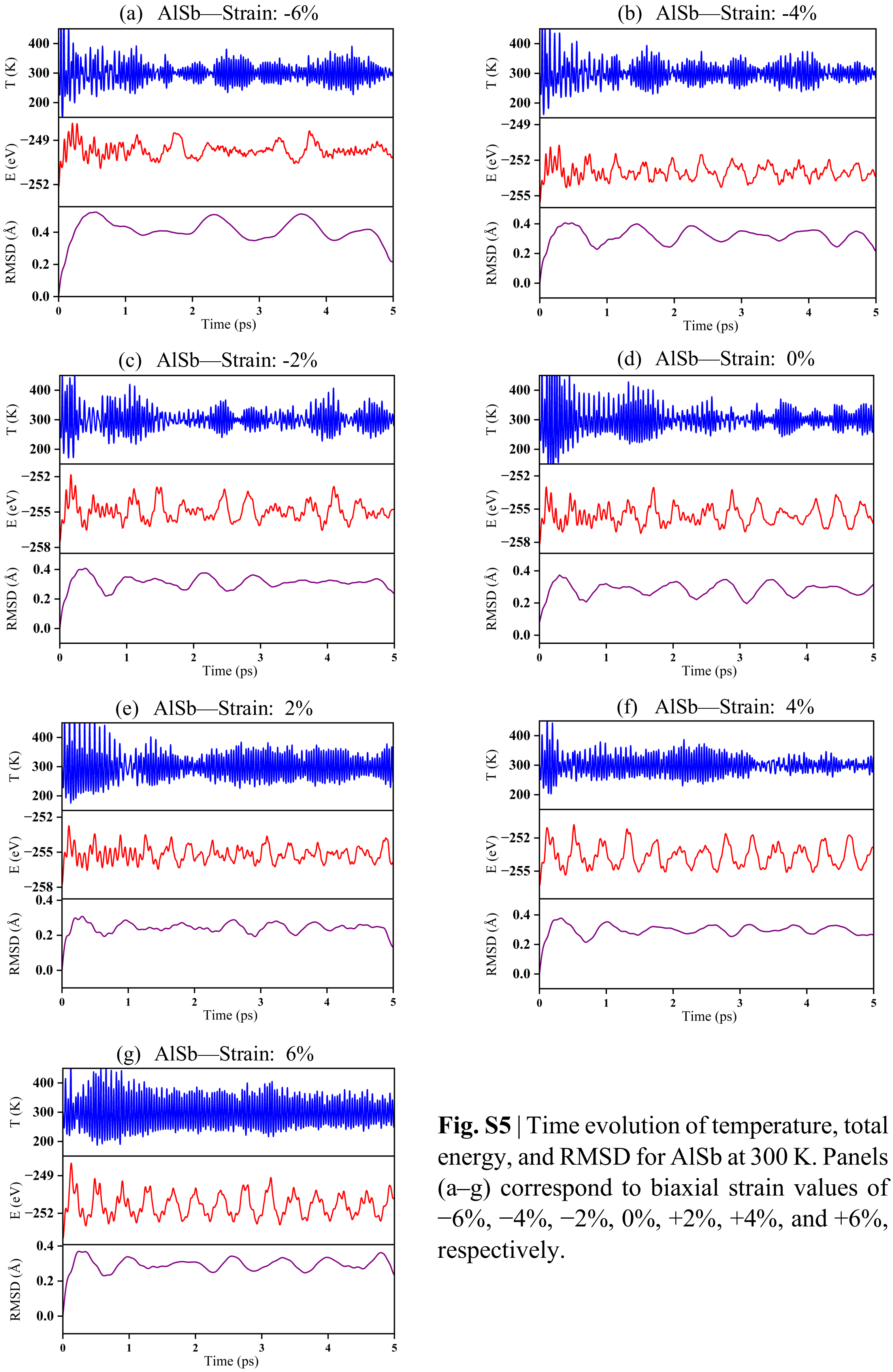

Fig. S5 | Time evolution of temperature, total energy, and RMSD for AlSb at 300 K. Panels (a–g) correspond to biaxial strain values of −6%, −4%, −2%, 0%, +2%, +4%, and +6%, respectively.

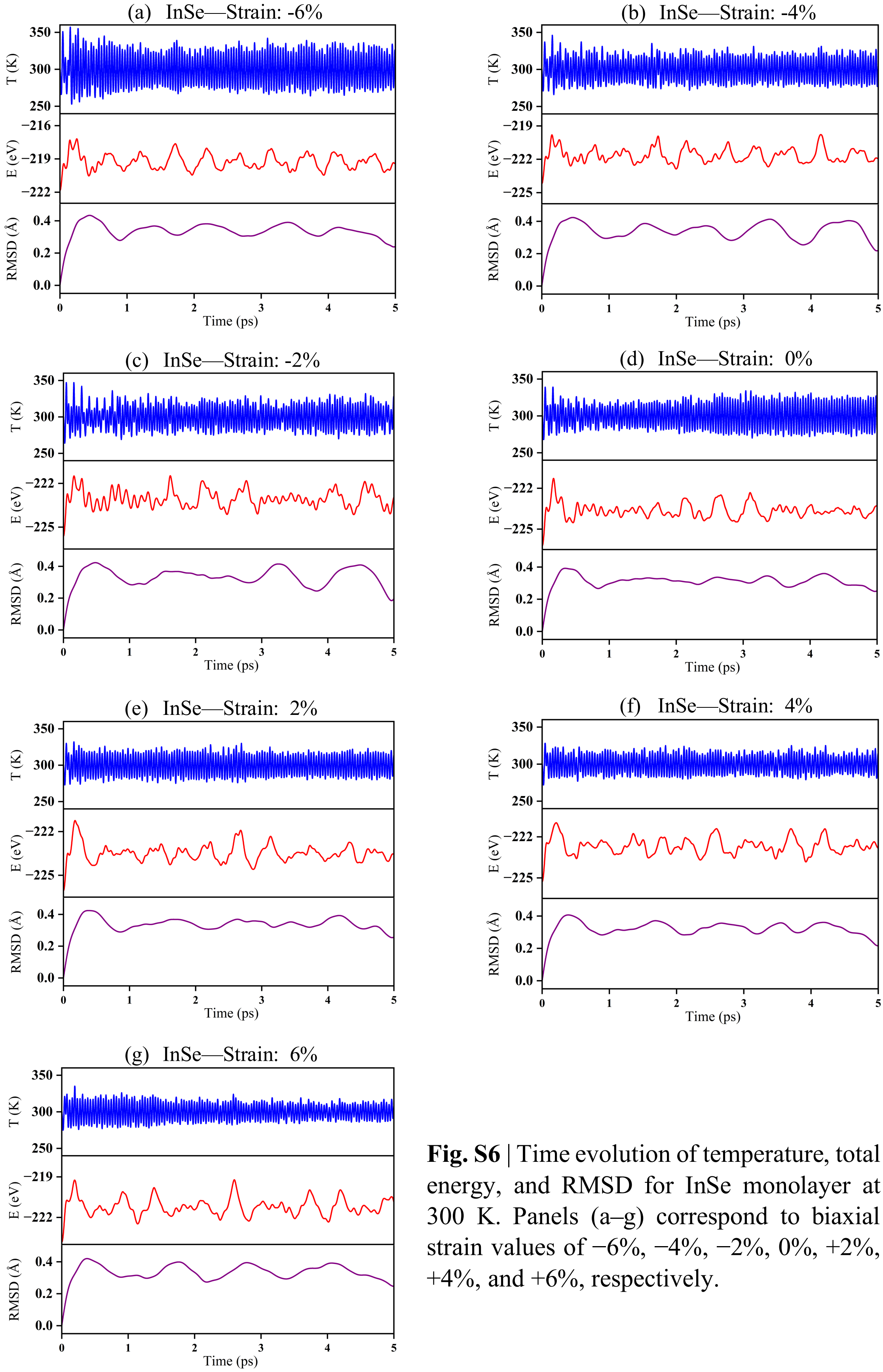

Fig. S6 | Time evolution of temperature, total energy, and RMSD for InSe monolayer at 300 K. Panels (a–g) correspond to biaxial strain values of −6%, −4%, −2%, 0%, +2%, +4%, and +6%, respectively.

Table S2 | Elastic constants C_{ij} of AlP, AlSb, and monolayer InSe calculated using PBE and HSE06 for biaxial strain from −6% to +6%.

Compound	C_{ij}	***Functional***	***Biaxial strain (%)***						
			-6.00	***-4.00***	***-2.00***	***0.00***	***2.00***	***4.00***	***6.00***
**AlP**	C_{11} (GPa)	HSE06	216.36	196.68	178.57	137.67	144.19	127.95	111.72
		PBE	206.79	188.08	170.54	131.41	137.47	121.79	106.79
	C_{12} (GPa)	HSE06	58.54	48.86	39.90	67.44	27.13	22.54	19.64
		PBE	59.78	49.64	40.64	66.55	27.11	22.28	18.46
	C_{13} (GPa)	HSE06	98.28	84.72	72.92	-	52.82	44.55	37.20
		PBE	97.32	83.85	71.99	-	52.02	43.78	36.49
	C_{33} (GPa)	HSE06	202.18	175.34	151.74	-	114.37	99.19	86.14
		PBE	194.56	168.18	145.23	-	108.63	93.93	81.27
	C_{44} (GPa)	HSE06	69.63	69.11	67.29	66.35	61.08	56.95	52.14
		PBE	64.48	64.48	63.18	62.31	57.72	53.98	49.54
	C_{66} (GPa)	HSE06	27.42	30.54	32.84	-	36.28	37.25	37.83
		PBE	24.93	28.04	30.30	-	33.59	34.55	35.12
**AlSb**	C_{11} (GPa)	HSE06	135.17	121.43	108.67	89.57	84.76	73.86	66.28
		PBE	128.95	115.77	103.65	76.16	81.15	70.60	60.86
	C_{12} (GPa)	HSE06	34.98	28.46	22.76	42.48	14.69	11.67	6.57
		PBE	37.82	31.01	24.90	35.90	15.90	12.81	10.03
	C_{13} (GPa)	HSE06	57.87	49.12	41.45	-	28.79	23.65	19.20
		PBE	59.23	50.39	42.69	-	29.95	24.81	20.29
	C_{33} (GPa)	HSE06	124.41	107.43	92.68	-	69.17	59.73	51.54
		PBE	119.80	103.09	88.59	-	65.59	56.41	48.53
	C_{44} (GPa)	HSE06	44.56	40.44	41.83	42.37	36.76	34.02	30.69
		PBE	39.72	39.28	38.10	36.30	34.18	31.80	28.87
	C_{66} (GPa)	HSE06	19.51	21.84	22.55	-	23.17	23.34	23.25
		PBE	16.99	18.52	19.50	-	20.68	20.84	20.77
**InSe**	C_{11} (N/m)	HSE06	77.93	69.40	61.62	58.83	48.22	43.01	37.60
		PBE	73.64	64.93	57.98	51.32	44.64	39.89	35.00
	C_{12} (N/m)	HSE06	23.72	20.41	17.12	15.98	12.36	10.16	8.69
		PBE	20.90	18.07	14.91	12.25	10.59	7.53	5.93
	C_{66} (N/m)	HSE06	27.11	24.49	22.25	21.43	17.93	16.43	14.46
		PBE	26.37	23.43	21.54	19.53	17.03	16.18	14.54

Table S3 | Strain-dependent elastic moduli *E*, *G*, *B*, and *ν* of AlP, AlSb, and monolayer InSe calculated using PBE and HSE06 for biaxial strain from −6% to +6%.

Compound	*Elastic Modulus*	*Functional*	*Biaxial strain (%)*						
			-6	*-4*	*-2*	*0*	*2*	*4*	*6*
**AlP**	*E* (GPa)	HSE06	143.28	139.33	133.27	129.72	117.94	108.96	99.27
		PBE	133.30	130.32	125.09	121.76	110.98	102.64	93.73
	G (GPa)	HSE06	54.60	53.92	52.35	51.40	47.74	44.76	41.34
		PBE	50.47	50.12	48.84	47.94	44.67	41.94	38.88
	B (GPa)	HSE06	126.98	111.61	97.80	90.85	74.24	64.23	55.25
		PBE	123.84	108.69	95.04	88.17	71.76	61.88	53.04
	ν	HSE06	0.312	0.292	0.273	0.262	0.235	0.217	0.201
		PBE	0.321	0.300	0.281	0.270	0.242	0.224	0.205
**AlSb**	*E* (GPa)	HSE06	93.20	87.52	84.34	84.26	71.89	65.57	59.74
		PBE	84.32	81.23	76.98	72.01	66.63	60.86	54.83
	G (GPa)	HSE06	35.87	34.12	33.54	33.47	29.50	27.37	25.47
		PBE	32.03	31.34	30.15	28.65	26.94	25.01	22.91
	B (GPa)	HSE06	77.27	67.05	57.92	58.17	42.58	36.15	30.44
		PBE	76.60	66.43	57.38	49.32	42.16	35.83	30.16
	ν	HSE06	0.299	0.282	0.257	0.259	0.219	0.198	0.173
		PBE	0.317	0.296	0.276	0.257	0.237	0.217	0.197
**InSe**	*E* (N/m)	HSE06	70.71	63.39	56.86	54.49	45.06	40.61	35.60
		PBE	67.71	59.89	54.15	48.39	42.13	38.47	33.99
	G (N/m)	HSE06	27.11	24.49	22.25	21.43	17.93	16.43	14.46
		PBE	26.37	23.43	21.54	19.53	17.03	16.18	14.54
	B (N/m)	HSE06	50.83	44.90	39.37	37.41	30.29	26.59	23.15
		PBE	47.27	41.50	36.45	31.78	27.61	23.71	20.46
	ν	HSE06	0.304	0.294	0.278	0.272	0.256	0.236	0.231
		PBE	0.284	0.278	0.257	0.239	0.237	0.189	0.169

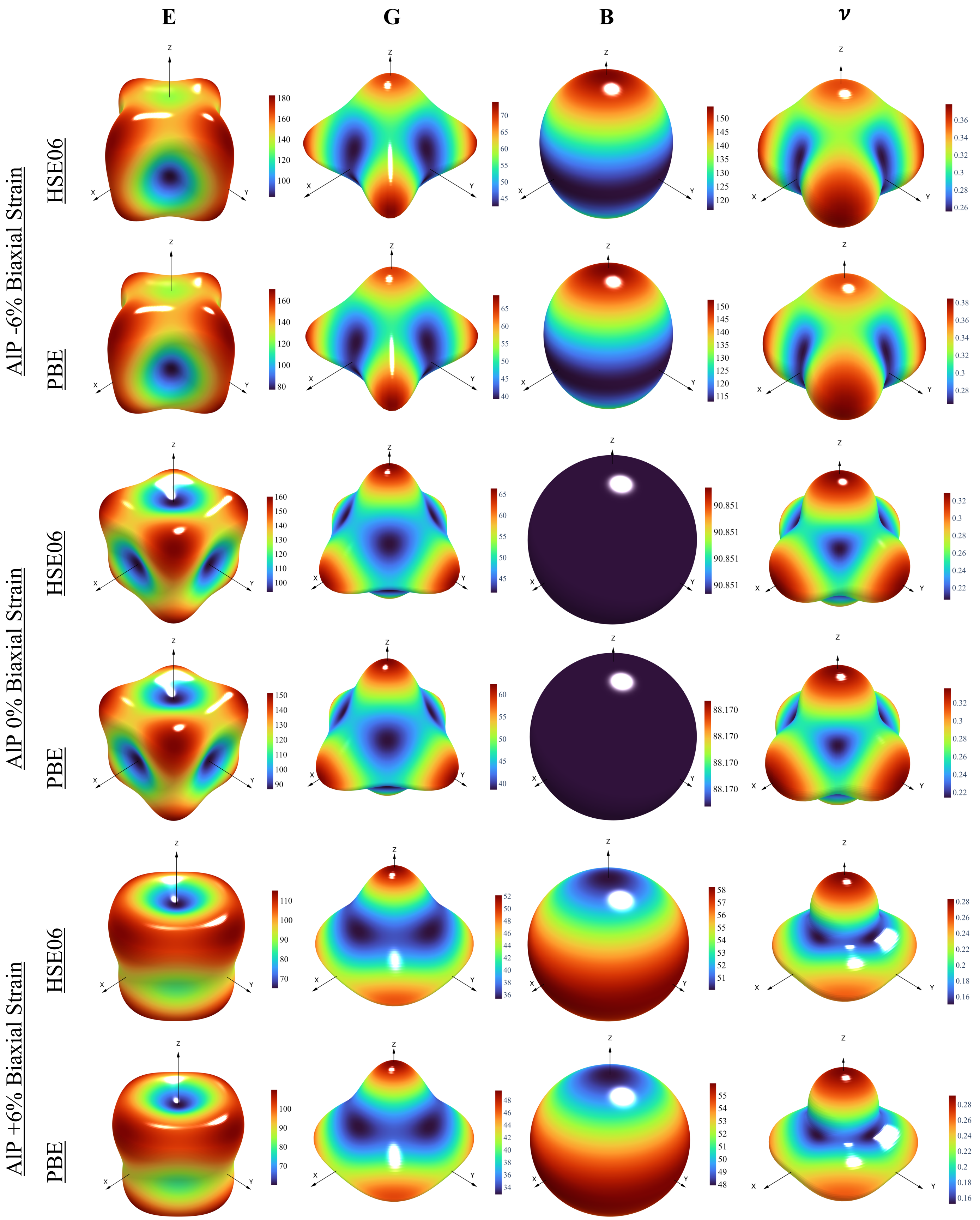

Fig. S7 | Directional elastic anisotropy of AlP at −6%, 0%, and +6% biaxial strain calculated using PBE and HSE06.

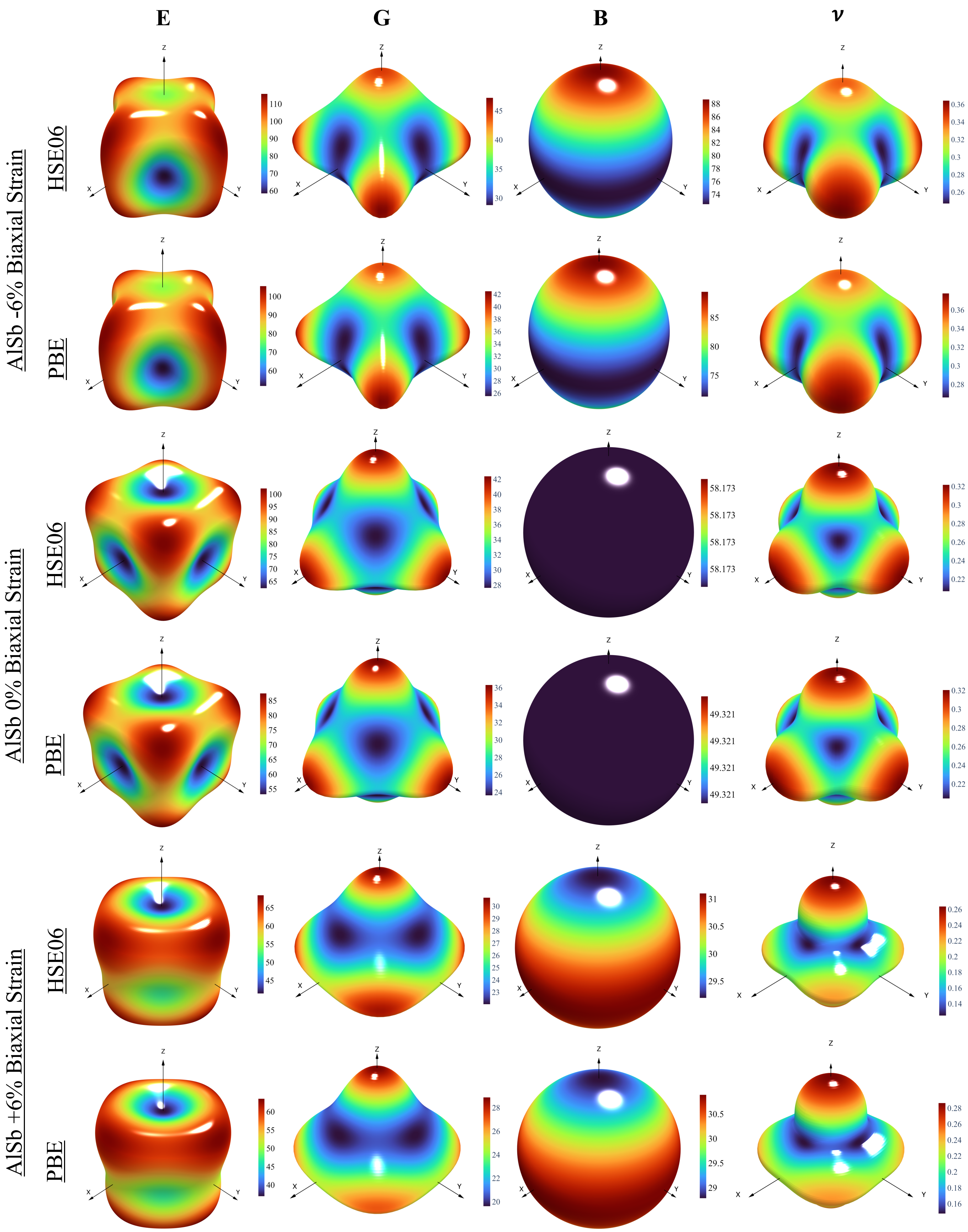

Fig. S8 | Directional elastic anisotropy of AlSb at −6%, 0%, and +6% biaxial strain calculated using PBE and HSE06.

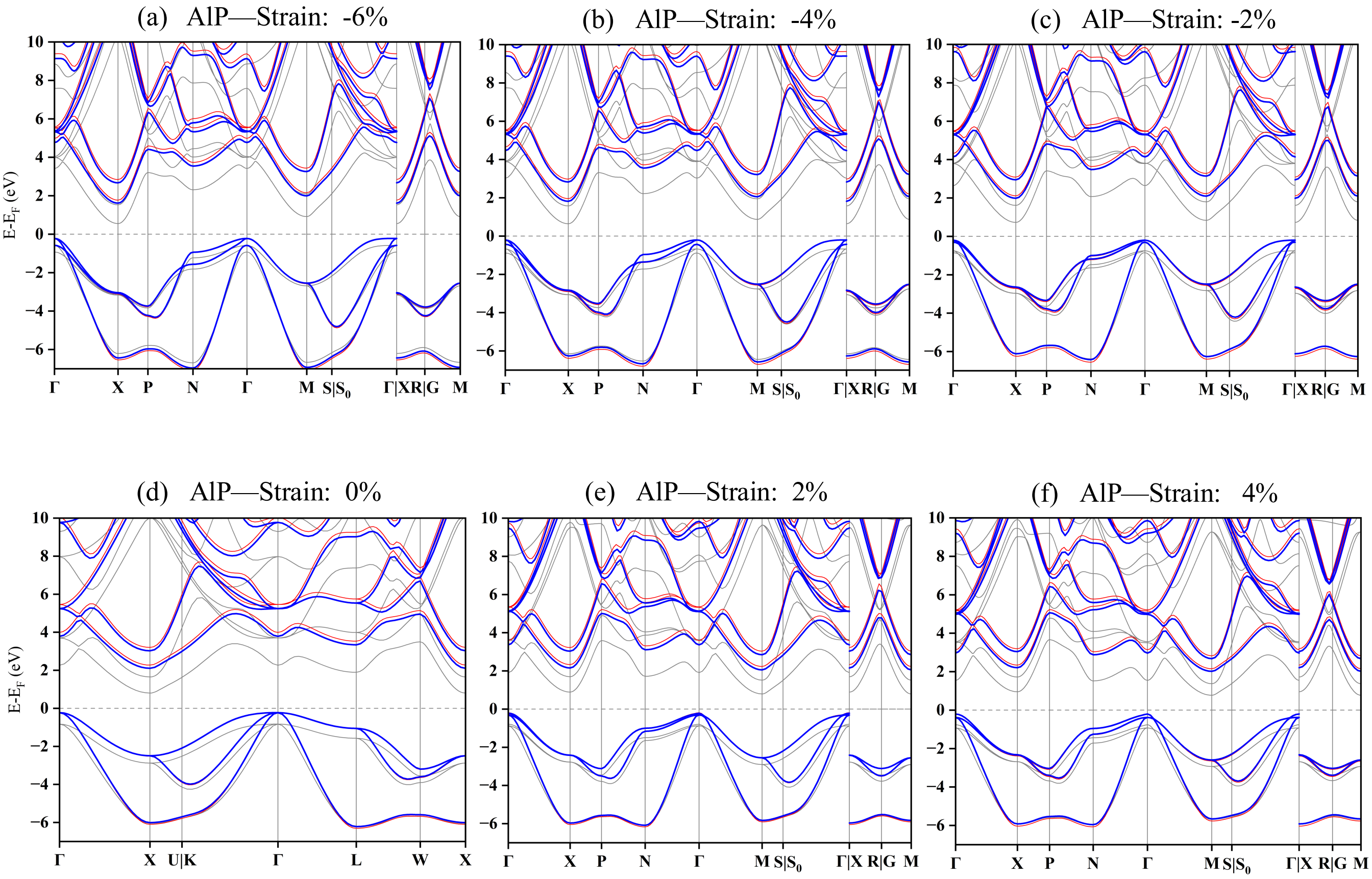

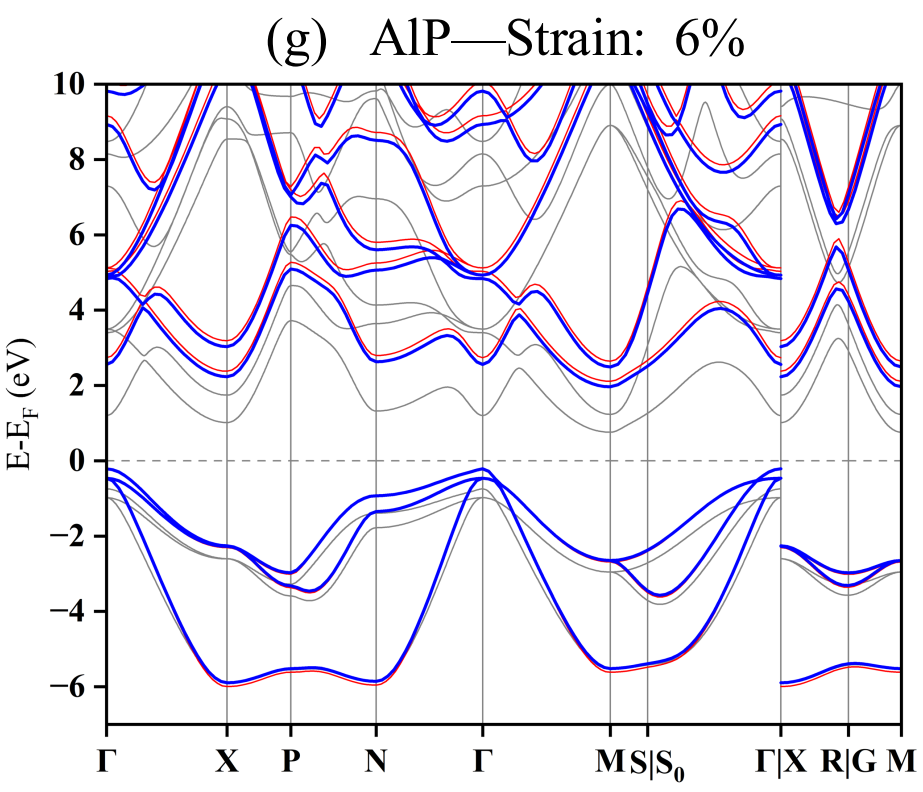

Fig. S9 | Electronic band structures of AlP under biaxial strain calculated using PBE (gray), HSE06 with 25% exact exchange (blue), and HSE06 with 30% exact exchange (red). Panels (a)–(g) correspond to strain values of −6%, −4%, −2%, 0%, +2%, +4%, and +6%, respectively.

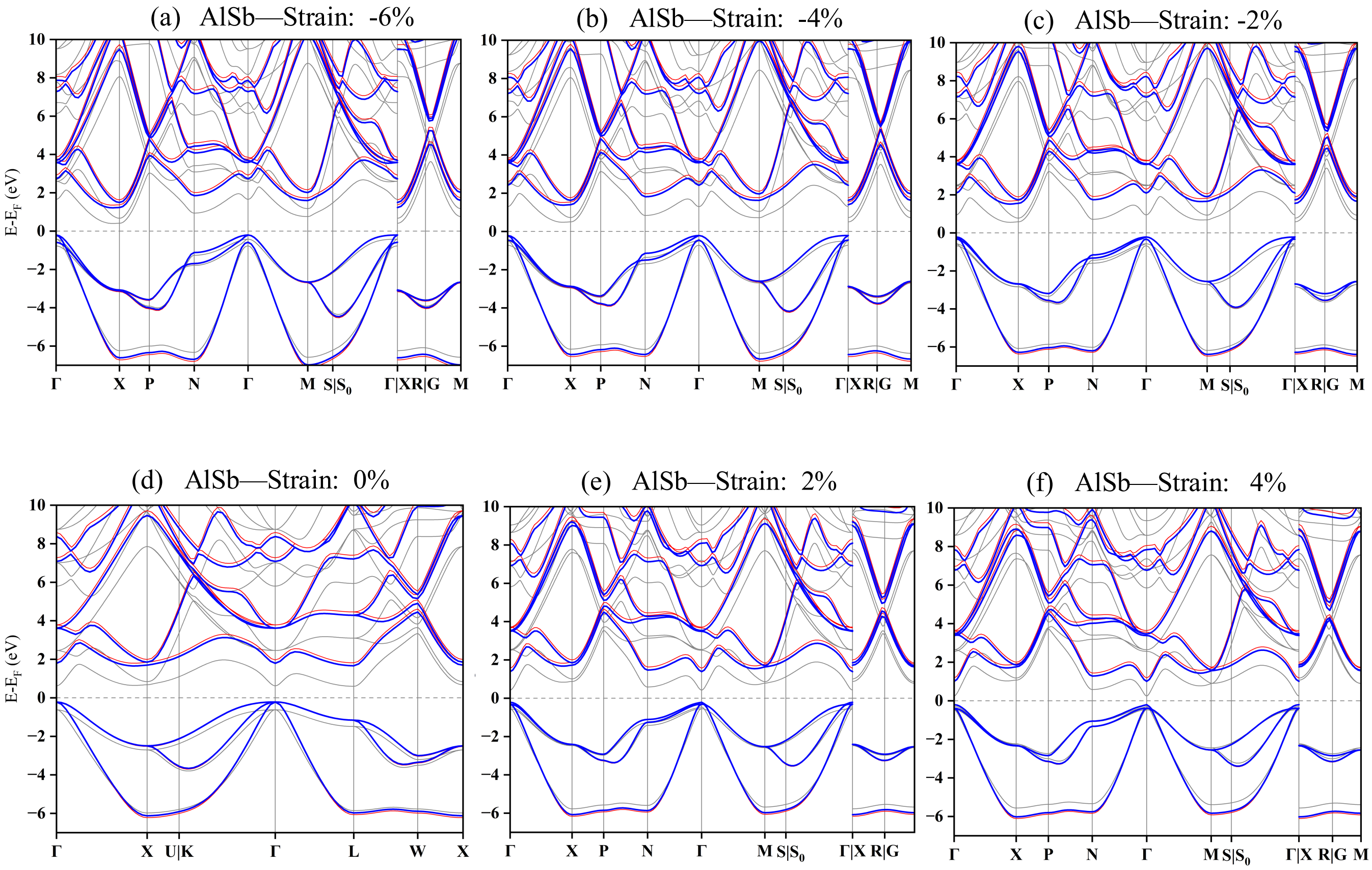

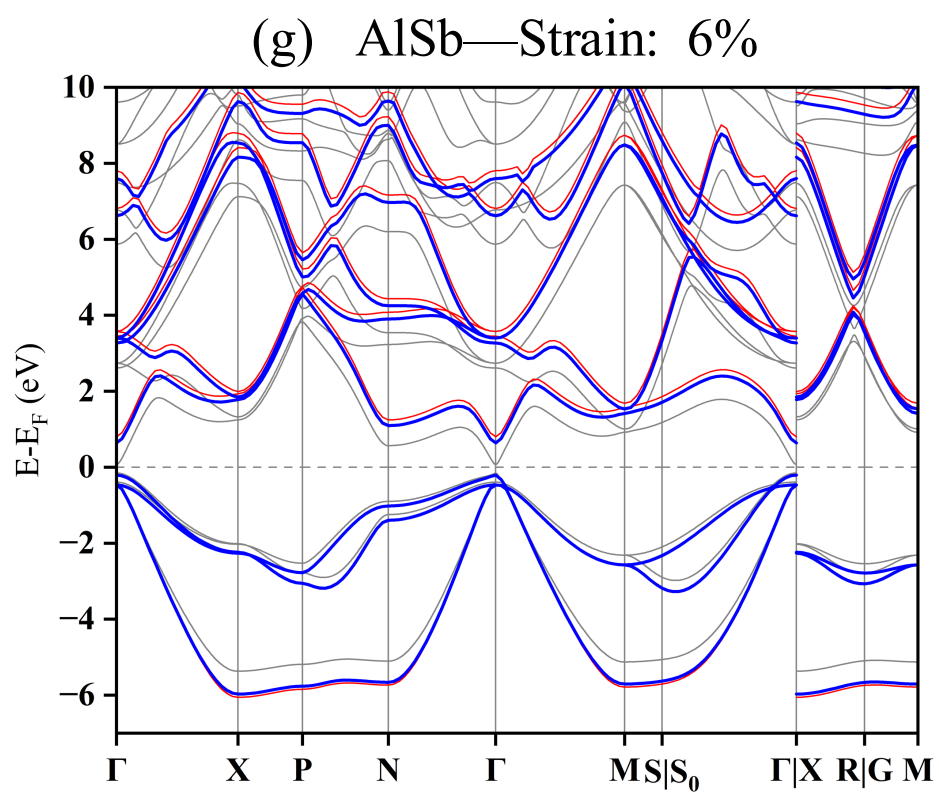

Fig. S10 | Electronic band structures of AlSb under biaxial strain calculated using PBE (gray), HSE06 with 25% exact exchange (blue), and HSE06 with 30% exact exchange (red). Panels (a)–(g) correspond to strain values of −6%, −4%, −2%, 0%, +2%, +4%, and +6%, respectively.

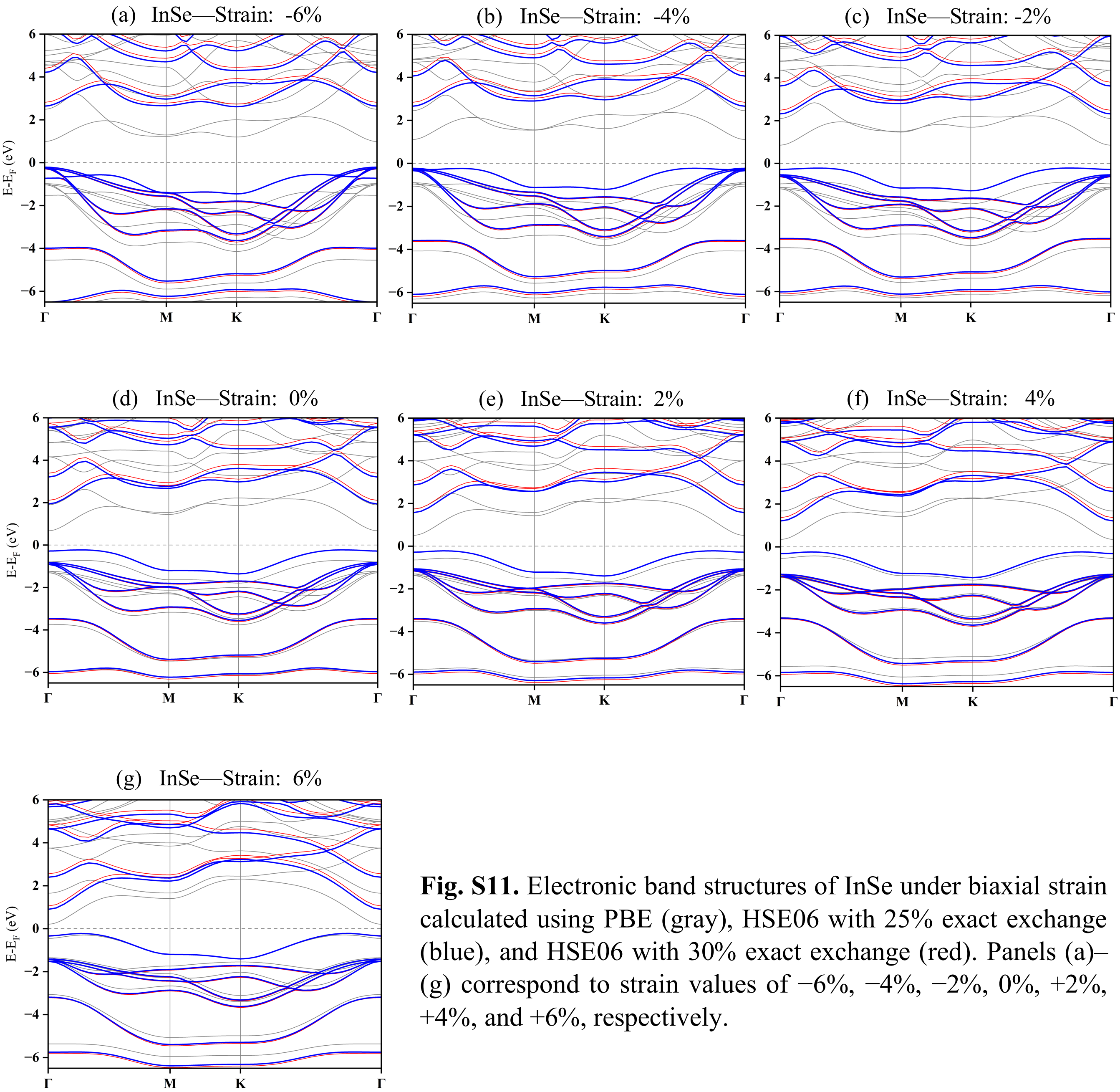

Fig. S11. Electronic band structures of InSe under biaxial strain calculated using PBE (gray), HSE06 with 25% exact exchange (blue), and HSE06 with 30% exact exchange (red). Panels (a)–(g) correspond to strain values of −6%, −4%, −2%, 0%, +2%, +4%, and +6%, respectively.

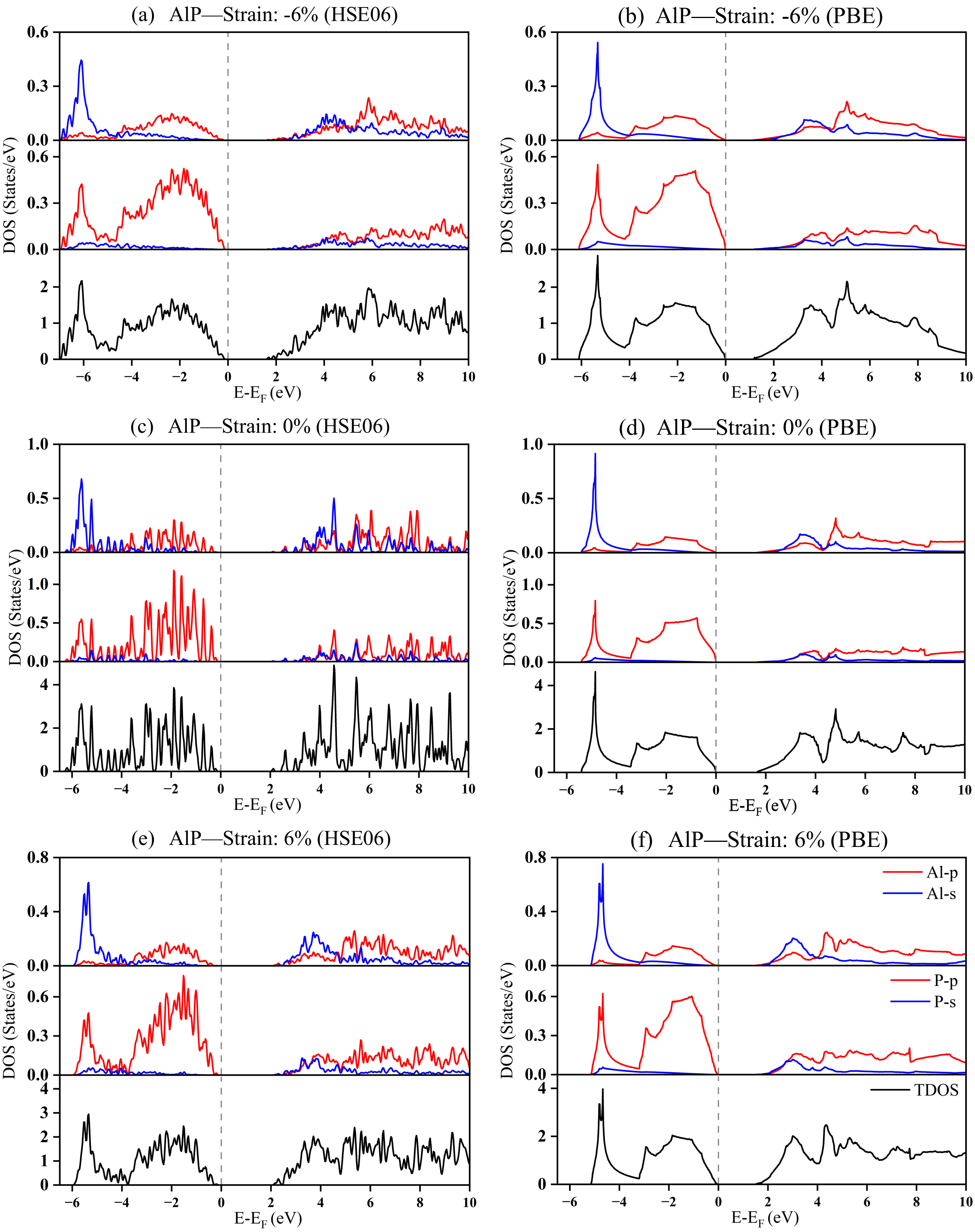

Fig. S12 | Total and orbital-projected density of states of bulk AlP under biaxial strain. Panels show −6%, 0%, and +6% strain calculated using the PBE and HSE06 functionals, as indicated. The energy axis is referenced to the Fermi level ($E - E_F = 0$ eV).

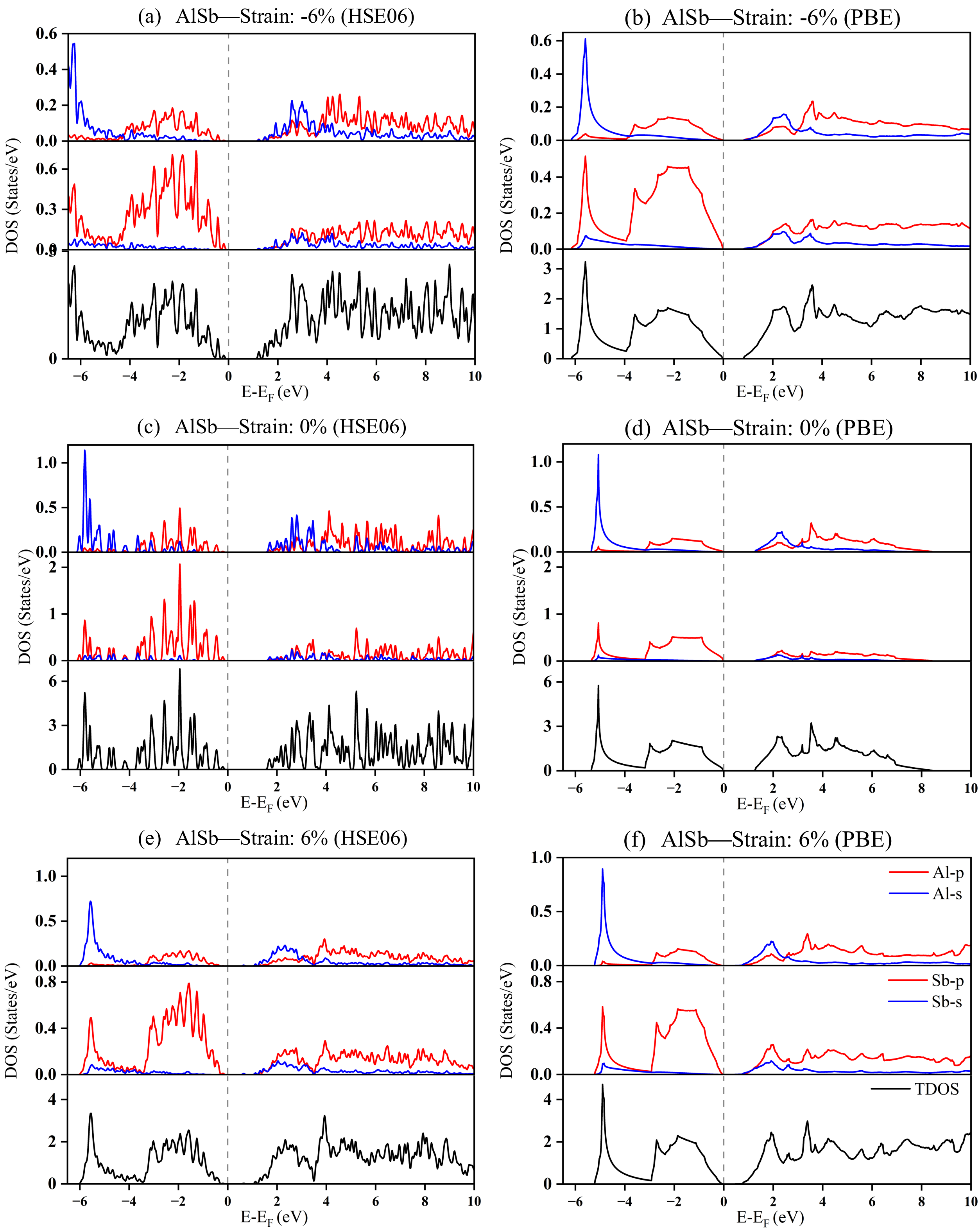

Fig. S13 | Total and orbital-projected density of states of bulk AlSb under biaxial strain. Panels show −6%, 0%, and +6% strain calculated using the PBE and HSE06 functionals, as indicated. The energy axis is referenced to the Fermi level ($E - E_F = 0$ eV).

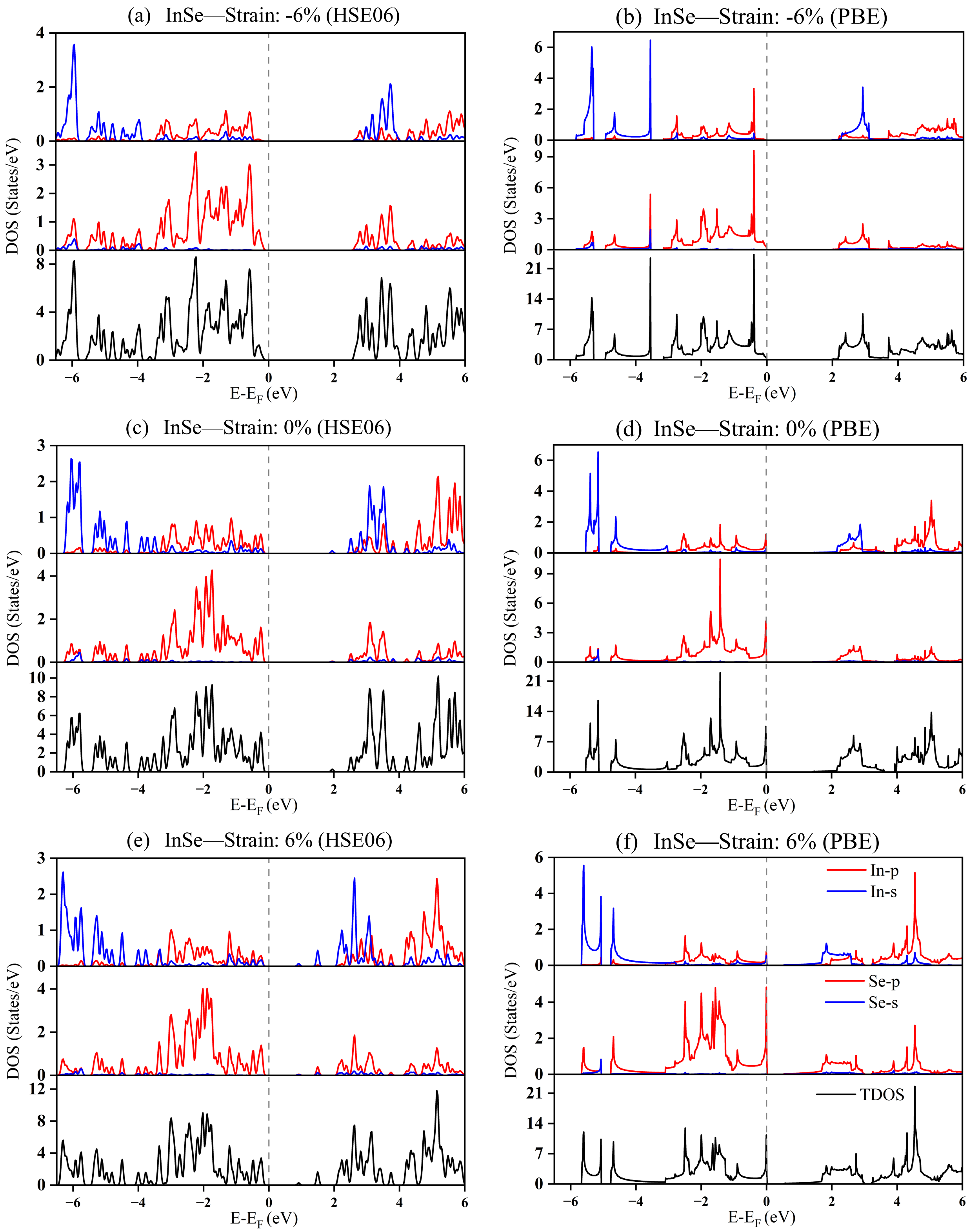

Fig. S14 | Total and orbital-projected density of states of InSe monolayer under biaxial strain. Panels show −6%, 0%, and +6% strain calculated using the PBE and HSE06 functionals, as indicated. The energy axis is referenced to the Fermi level ($E - E_F = 0$ eV).

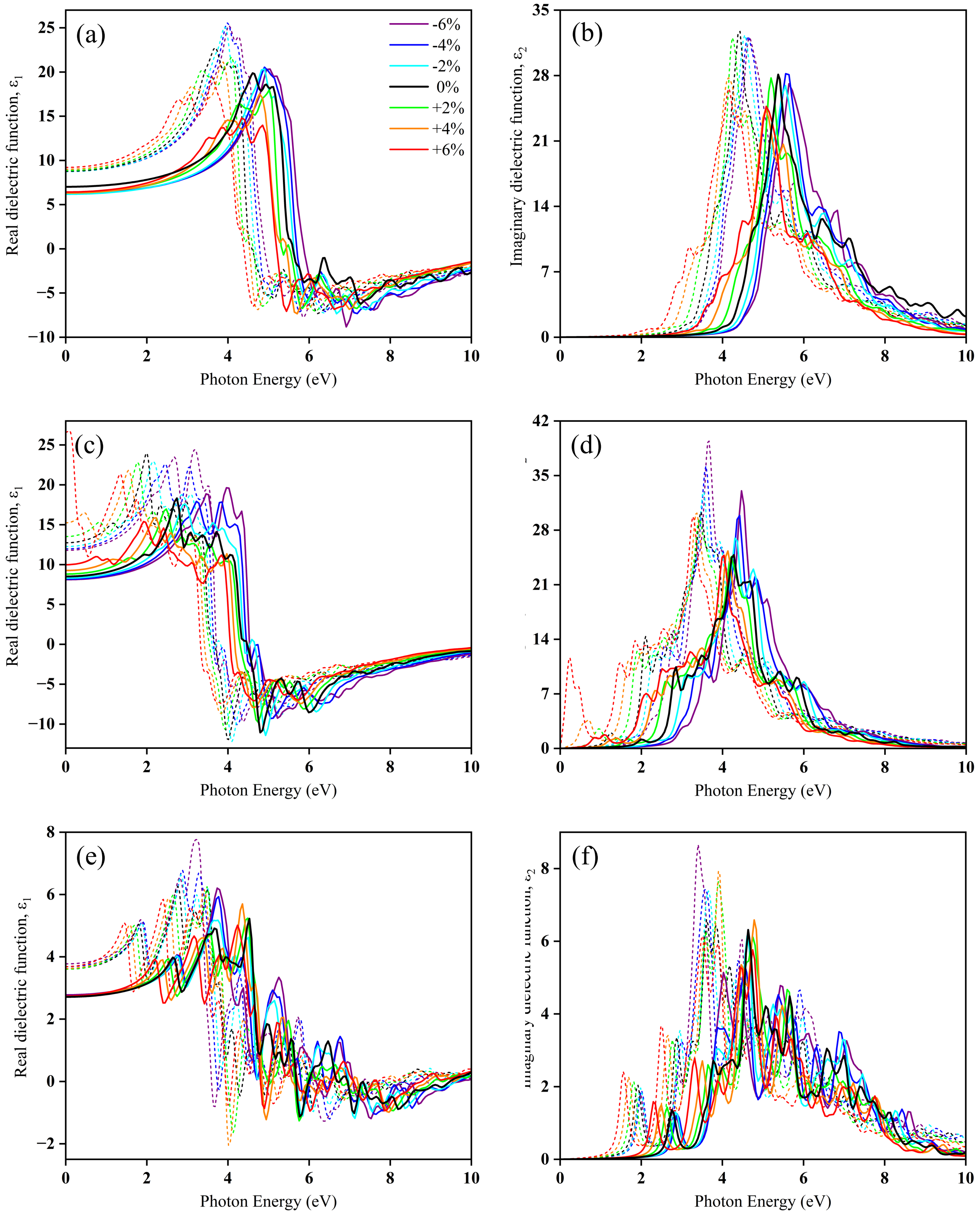

Fig. S15 | Frequency-dependent real and imaginary parts of the dielectric function of bulk AlP, bulk AlSb, and monolayer InSe under biaxial strain. Panels (a), (c), and (e) show the real dielectric function (ε_1) of bulk AlP, bulk AlSb, and monolayer InSe, respectively, while panels (b), (d), and (f) show the corresponding imaginary dielectric function (ε_2). Solid and dashed lines represent the HSE06 and PBE results, respectively.

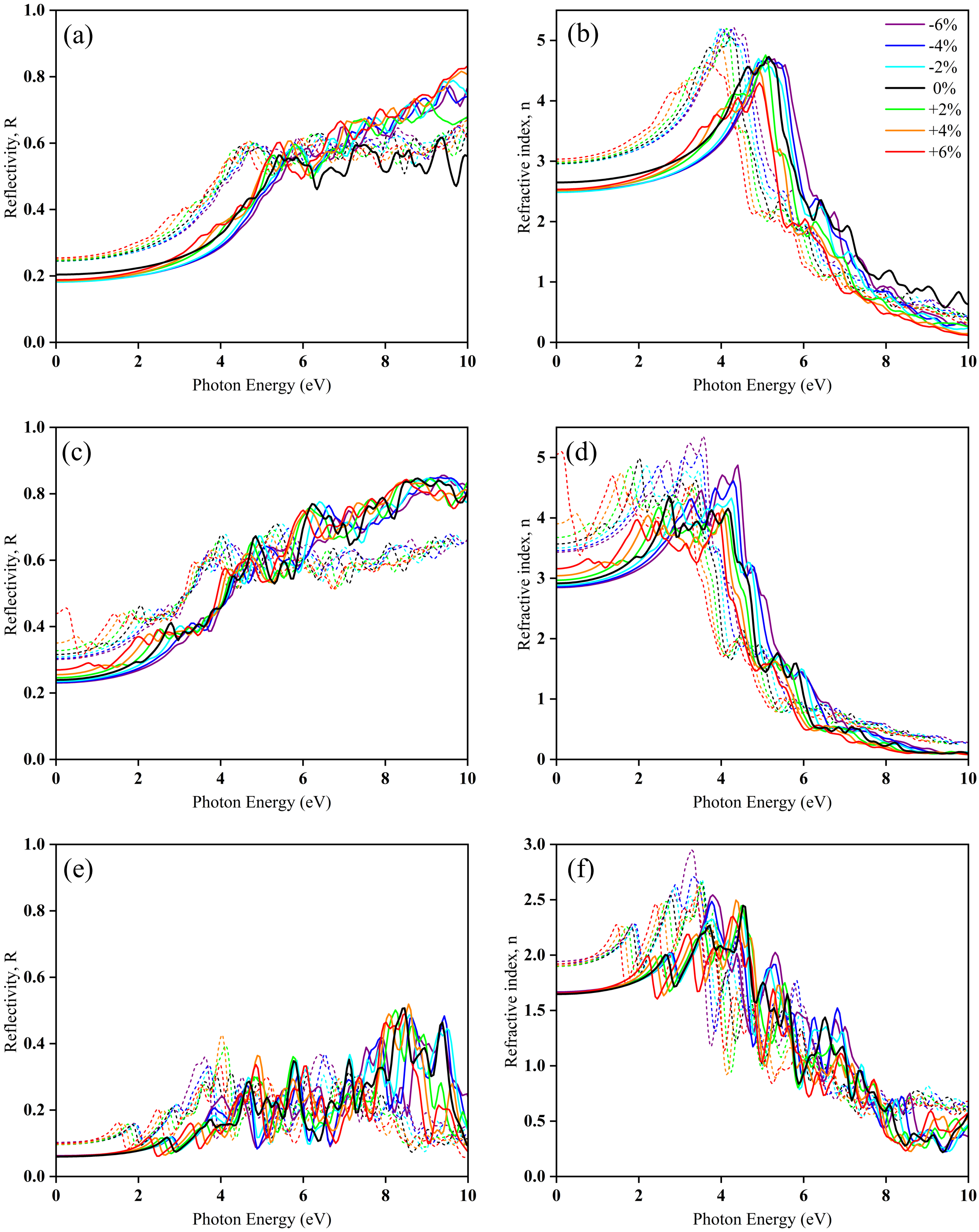

Fig. S16 | Frequency-dependent reflectivity and refractive index of bulk AlP, bulk AlSb, and monolayer InSe under biaxial strain. Panels (a), (c), and (e) show the reflectivity (R) of bulk AlP, bulk AlSb, and monolayer InSe, respectively, while panels (b), (d), and (f) show the corresponding refractive index (n). Solid and dashed lines represent the HSE06 and PBE results, respectively.

Automated extraction of the optical onset

To obtain a reproducible estimate of the optical absorption threshold, the optical onset was extracted automatically from the calculated absorption spectra using a Python script developed in this work. The absorption spectra were first smoothed using a Gaussian filter to suppress numerical noise while preserving the low-energy absorption edge. The first bright excitonic peak was subsequently identified from the smoothed spectrum, and the fitting region was restricted to the absorption edge preceding this peak to exclude higher-energy inter-band transitions. Within this region, the optimal linear fitting interval was selected by maximizing the coefficient of determination (R^2). The optical onset energy was then determined from the intercept of the corresponding linear regression with the energy axis.

The automated procedure provides a consistent and objective determination of the optical onset by eliminating subjective selection of the fitting region. The same fitting parameters and extraction criteria were applied uniformly to all materials and strain states. Fig. S17 presents a representative example of the extraction procedure for unstrained bulk AlP, including the absorption spectrum, the automatically selected fitting region, the optimal linear regression, the extracted optical onset, the first excitonic peak, and the corresponding fitting statistics.

The Python implementation used for the automated optical-onset extraction is publicly available through Zenodo (DOI: https://doi.org/10.5281/zenodo.21635897), enabling full reproducibility of the reported optical-onset analysis.

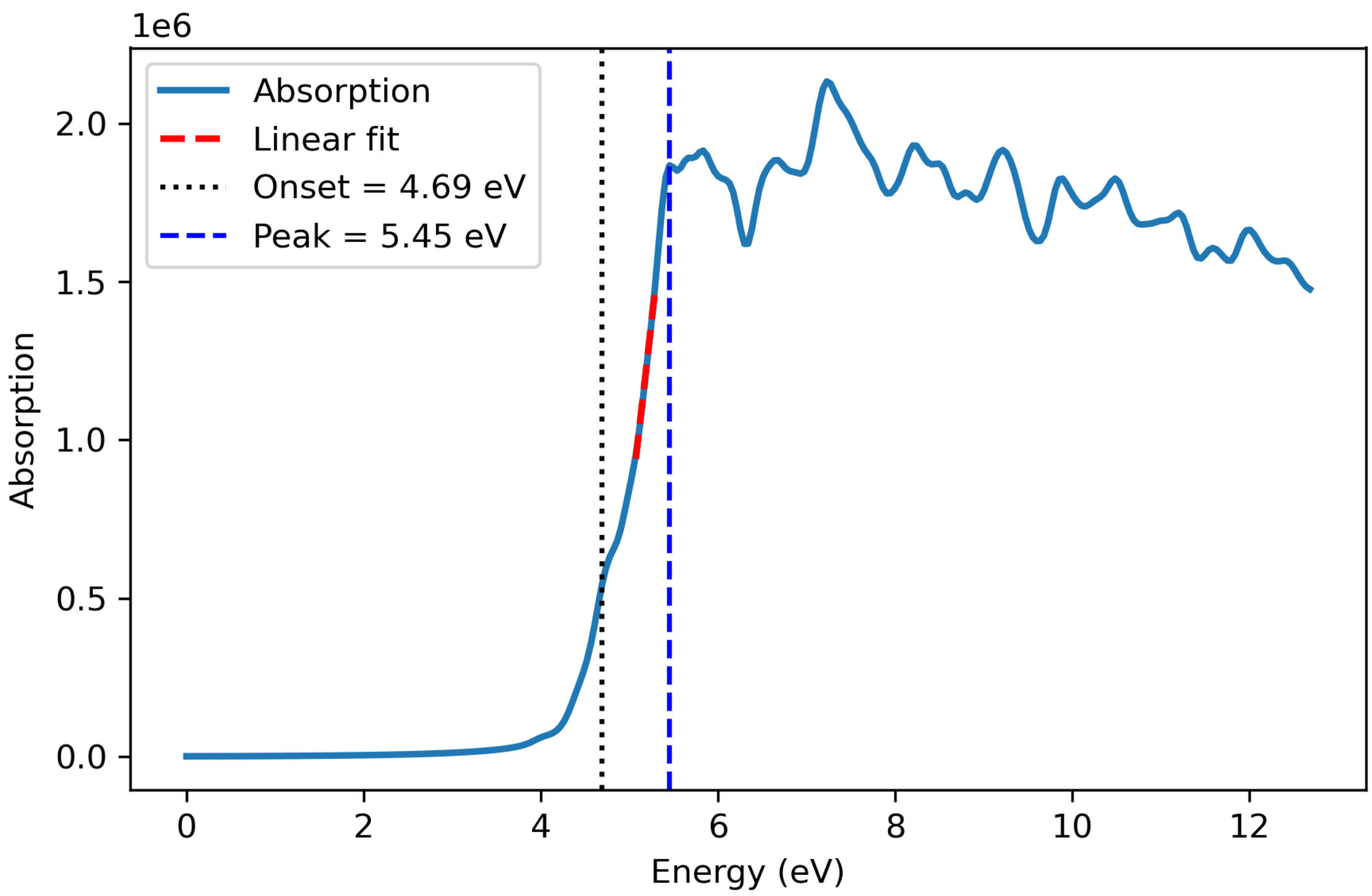

Fig. S17. Representative example of the automated optical-onset extraction procedure from the absorption spectrum of unstrained AlP.
